# MERISTEM-DEFECTIVE regulates the balance between stemness and differentiation in the root meristem through RNA splicing control

**DOI:** 10.1242/dev.201476

**Published:** 2023-04-05

**Authors:** Helen L. Thompson, Weiran Shen, Rodrigo Matus, Medhavi Kakkar, Carl Jones, David Dolan, Sushma Grellscheid, Xiyan Yang, Na Zhang, Sina Mozaffari-Jovin, Chunli Chen, Xianlong Zhang, Jennifer F. Topping, Keith Lindsey

**Affiliations:** ^1^ Department of Biosciences, Durham University, Durham DH1 3LE, UK; ^2^ National Key Laboratory of Crop Genetic Improvement, Huazhong Agricultural University, Wuhan 430070, China; ^3^ College of Life Science and Technology, Huazhong Agricultural University, Wuhan 430070, China; ^4^ Department of Cellular Biochemistry, Max Planck Institute of Biophysical Chemistry, 37077 Goettingen, Germany

**Keywords:** *Arabidopsis*, Meristem function, MDF/DOT2, RNA splicing, RSZ33, ACC1, SR34

## Abstract

Plants respond to environmental stresses through controlled stem cell maintenance and meristem activity. One level of gene regulation is RNA alternative splicing. However, the mechanistic link between stress, meristem function and RNA splicing is poorly understood. The *MERISTEM-DEFECTIVE* (*MDF*) *Arabidopsis* gene encodes an SR-related family protein, required for meristem function and leaf vascularization, and is the likely orthologue of the human SART1 and yeast Snu66 splicing factors. MDF is required for the correct splicing and expression of key transcripts associated with root meristem function. We identified *RSZ33* and *ACC1*, both known to regulate cell patterning, as splicing targets required for MDF function in the meristem. *MDF* expression is modulated by osmotic and cold stress, associated with differential splicing and specific isoform accumulation and shuttling between nucleus and cytosol, and acts in part via a splicing target *SR34*. We propose a model in which MDF controls splicing in the root meristem to promote stemness and to repress stress response, cell differentiation and cell death pathways.

## Teaser

**Summary:** The protein MERISTEM-DEFECTIVE regulates *Arabidopsis* meristem function through its role as a splicing factor, mediated through splicing targets RSZ33, ACC1 and SR34.

## INTRODUCTION

There is much interest in understanding the molecular mechanisms regulating meristem function, and interacting networks involving hormonal crosstalk and regulatory transcription factors have been identified (Santuari et al., 2016). Less well understood are the mechanisms determining how stress responses impact the balanced relationship between the stemness of meristems and cell differentiation processes, in order to control growth and development.

There is currently a large research effort into the role of RNA processing [microRNAs; long non-coding RNAs; nonsense-mediated mRNA decay (NMD); alternative splicing (AS)] in the control of plant development and stress responses (Tanabe et al., 2006; Duque, 2011; Chen et al., 2013). There is good evidence for the role of AS in plant development (Szakonyi and Duque, 2018) and in plant responses to stress (Duque, 2011), including temperature stress (Capovilla et al., 2018; Huertas et al., 2019), salt and nutrient stress (Lee et al., 2006; Nishida et al., 2017), and responses to abscisic acid (ABA; Cruz et al., 2014; Zhu et al., 2017) or other environmental cues, such as circadian rhythm (Cui et al., 2017). However, although more than 300 genes encoding proteins putatively involved in splicing have been identified in *Arabidopsis* by homology searching (Reddy et al., 2013), very few have been tested experimentally for tissue-specific activities or in relation to the control of gene expression in response to external factors.

**Fig. 1.**
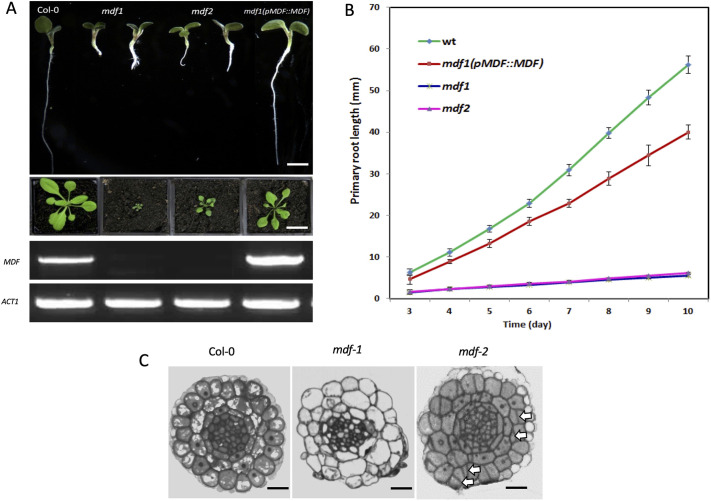
***mdf* mutants show defective cell division and growth*.*** (A) Top: 10 dpg seedlings of the two T-DNA mutants, *mdf-1* and *mdf-2*, wild-type Col-0 and a complemented *mdf-1*×*pMDF::MDF* line. Scale bar: 0.5 cm. Middle: Phenotypes of wild-type Col-0, *mdf-1*, *mdf-2* and the complemented *pMDF::MDF* at 21 dpg. Scale bar: 1.5 cm. Bottom: Semi-quantitative RT-PCR of *MDF* in wild type, both mutants and the complemented line. The full-length transcript was absent in both mutants. Control PCR with *ACT1* primers shown below. (B) Change in primary root length over time for wild-type Col-0 (wt), *mdf-1* and *mdf-2* mutants and the complemented line (*pMDF::MDF*). Data are mean±s.e.m., *n*=5. (C) Transverse sections through the root tip of wild-type Col-0, *mdf-1* and *mdf-2* mutants of seedlings at 7 dpg, and at approximately equivalent positions in the meristem above the quiescent centre (although the cellular organization of wild-type and *mdf* roots is very different). *mdf-2* cells are more organised than in *mdf-1*, but show evidence of irregular divisions (arrows indicate some examples). Scale bars: 10 µm.

We previously identified the *MDF* gene (At5g16870) in an expression screen in the developing *Arabidopsis* embryonic root (Casson et al., 2005). It encodes a predicted arginine-serine (RS) domain protein, with homology to the human SART1 and yeast Snu66 proteins, which are splicing factors (Casson et al., 2009; Fig. S1). *MDF* is expressed at relatively high levels in the embryonic root meristem, and subsequently in the seedling root and shoot meristems, with lower levels of expression in vascular tissues (Fig. S1; Casson et al., 2005, 2009). It was also identified separately in a screen for vascular tissue-defective mutants, and given the name *DEFECTIVELY ORGANIZED TRIBUTARIES* (*DOT2*; Petricka et al., 2008). *mdf/dot2* loss-of-function mutants are dwarfed with short hypocotyls and roots, and occasionally three cotyledons (Casson et al., 2009). Some mutant individuals are seedling lethal (Casson et al., 2009), but those that develop leaves have smaller aerial parts than wild type, with *mdf-1* showing a more severe phenotype than *mdf-2.* Defects are largely rescued in the complementation line (Fig. 1A,B; Fig. S2). A very recent paper has shown that MDF is a likely splicing factor with a similar function to hSART1 and mediates responses to DNA damage (de Luxan-Hernandez et al., 2022). Here, we investigate the role of MDF in meristem function, determine whether it is itself spliced, and identify downstream targets linked to meristem function.

## RESULTS AND DISCUSSION

### MDF is a component of the plant spliceosome

The predicted 820 amino acid MDF protein (Fig. S1) shares 41% identity with hSART1 (Fig. S1D). hSART1 and the yeast homologue Snu66 are key components of the maturing spliceosome, involved in recruiting the tri-snRP during B complex formation, with secondary roles in cell cycle control (Makarova et al., 2001). Modelling of the putative three-dimensional structure of the MDF protein suggests a strong structural similarity with hSART1 (Fig. S2A). de Luxan-Hernandez et al. (2022) observed interaction between MDF and the spliceosome component STABILIZED 1 (STA1; the plant homologue of yeast PRP6), although interaction with another tri-snRNP complex protein, LSM8, was not detected. To investigate further the possible molecular relationship between MDF and nuclear proteins in *Arabidopsis*, we carried out protein–protein interaction studies with the putative homologous plant spliceosome components BRR2A and PRP8A and the nuclear protein BIN2, a nuclear-localised kinase required for root meristem maintenance that interacts with the nuclear protein BZR1, which is required for root development. Although we found no detectable interaction between MDF and PRP8A in yeast two-hybrid experiments (Fig. S2B) or by bimolecular fluorescence complementation (BiFC), we observed colocalization and BiFC interaction with BRR2A, a splicing factor conserved in eukaryotes (Fig. S2C-I; Mahrez et al., 2016); and with both BIN2 and BZR1, which also interact with each other (Fig. S2F-H; Yan et al., 2009; Li et al., 2020). In agreement with de Luxan-Hernandez et al. (2022), these data strongly support the view that MDF is a nuclear protein and a structural component of the plant spliceosome complex.

### MDF promotes stemness and represses differentiation pathways

A role for MDF in splicing was further confirmed by RNA sequencing (RNA-Seq) on two independent *mdf* loss-of-function mutants (*mdf-1* and *mdf-2*; Casson et al., 2009; de Luxan-Hernandez et al., 2022) compared with wild type. RNA-Seq analysis provided additional information on the biological pathways in which MDF plays a role, and also allowed comparison of patterns of splicing isoform profiles compared with a wild-type control by analysing the data output from rMATS for AS events (Shen et al., 2014). We hypothesised that, if MDF acts as an essential splicing factor, we would see both altered patterns of RNA isoforms for some genes, and changes in the abundance of other, non-spliced, transcripts that may be targets of mis-spliced regulatory RNAs.

Alternative splicing analysis was initially performed by comparing the RNA-Seq data of *mdf-1* (which showed more transcriptional changes than *mdf-2*) with the *Arabidopsis* reference transcriptome AtRTD2 (Zhang et al., 2017; Fig. S3A). Gene enrichment analysis of the 2015 alternative splicing events identified by rMATS analysis showed that the most common pathways affected were ‘mRNA metabolic process’, ‘vegetative to reproductive phase transition of meristem’ and ‘RNA splicing’ (*P*<0.05) (Fig. S3B). This analysis identified 4706 genes that were spliced differentially compared with wild type with a false detection rate (FDR) of 0.01 and a 10% inclusion difference minimum (Thompson et al., 2023; https://doi.org/10.5061/dryad.b2rbnzskc). The most frequently detected mis-splicing events in *mdf-1* were retained introns, both increased (1028 events) and decreased (1597 events) frequencies compared with wild type, followed by alternative 5′ splice site use (477 increased, 333 decreased events) and alternative 3′ splice site use (412 increased, 451 decreased events), skipped exons (279 increased, 110 decreased events), and least frequent were other, multiple exon events (11 increased, eight decreased events) (Fig. S3C).

de Luxan-Hernandez et al. (2022) found upregulation of stress-related genes and downregulation of cell division genes, among others. We found that 4195 genes were upregulated and 5404 genes downregulated in *mdf-1*, and 2830 were upregulated and 3449 downregulated in *mdf-2*, which has a less severe mutant phenotype after the seedling stage (Fig. 1A, Figs S3A, S4A,B). Unbiased gene ontology (GO) enrichment analysis was carried out and treemaps were generated using data from REVIGO (Supek et al., 2011). Both *mdf-1* and *mdf-2* transcriptomes exhibited similar patterns of upregulation of stress-related genes (Fig. S3D-H), and enriched GO terms included ‘response to stress’, ‘defence response’, ‘response to other organism’, ‘immune response’, ‘reactive oxygen species metabolism’ and ‘response to ethylene’. In both tree maps, ‘aging’ and ‘programmed cell death’ were also shown to be significantly upregulated. This suggests that MDF plays a negative regulatory role in different stress response pathways, with the stronger (*mdf-1*) allele affecting more genes in these pathways.

The majority of the downregulated GO terms are related to signalling and development, including ‘auxin signalling pathway’, ‘protein phosphorylation’ and ‘tissue development and growth’. We previously showed altered auxin distribution in *mdf* mutant root tips, associated with defective PIN protein localization [which is dependent on correct *PLETHORA* (*PLT*) gene expression; Casson et al., 2009; Santuari et al., 2016; Aida et al., 2004] and potentially accounting for misexpression of auxin-regulated *WUSCHEL-RELATED HOMEOBOX5* (*WOX5*; Sarkar et al., 2007). Our RNA-Seq data showed that the expression of several known root meristem regulation genes, including PIN-FORMED genes (*PIN1* to *PIN7*), *PLT1*, *PLT2* and *PLT4*, *WOX1*, *WOX4* and *WOX5*, *SHORTROOT* (*SHR*) and *POLARIS*, was significantly downregulated in *mdf-1*, and most also in the less severe *mdf-2*, whereas *PLT5* was upregulated (Fig. S3B,I; Tables S1, S2). The molecular reason for the weaker phenotype of the *mdf-2* allele versus *mdf-1* is currently unclear, but it was evident in both transcriptional analysis as well as seedling phenotype during vegetative growth (Fig. 1A,B). The radial pattern of the *mdf* primary root was abnormal (Fig. 1C), with similar supernumerary cell divisions to those seen in the *shr* mutant (Helariutta et al., 2000). Therefore, MDF function is required for the correct expression and biological activity of a number of essential meristem genes. The PLT and SHR/SCR pathways, which contribute to stem cell niche formation, function independently (Aida et al., 2004), but our results show that both are regulated by the MDF pathway. These transcriptome data are in agreement with RT-qPCR analysis of PLT, PIN, SCR and SHR genes in *mdf-1* (Table S1, Fig. S4; Casson et al., 2009), and a corresponding reduction of expression of cell cycle-related genes and upregulation of stress-responsive genes revealed by RT-qPCR (Fig. S4) further validated the RNA-Seq data. The RNA-Seq analysis, in the context of the phenotypic analysis, therefore suggests a role for MDF in promoting auxin-mediated and other signalling pathways to promote meristem function and stemness, while suppressing pathways associated with stress response and differentiation pathways.

These results are consistent with MDF being required for correct meristem gene expression control via splicing, and we aimed to identify other root meristem genes that may be spliced by an MDF-dependent mechanism.

### MDF controls meristem activity through RSZ33 and ACC1

**Fig. 2.**
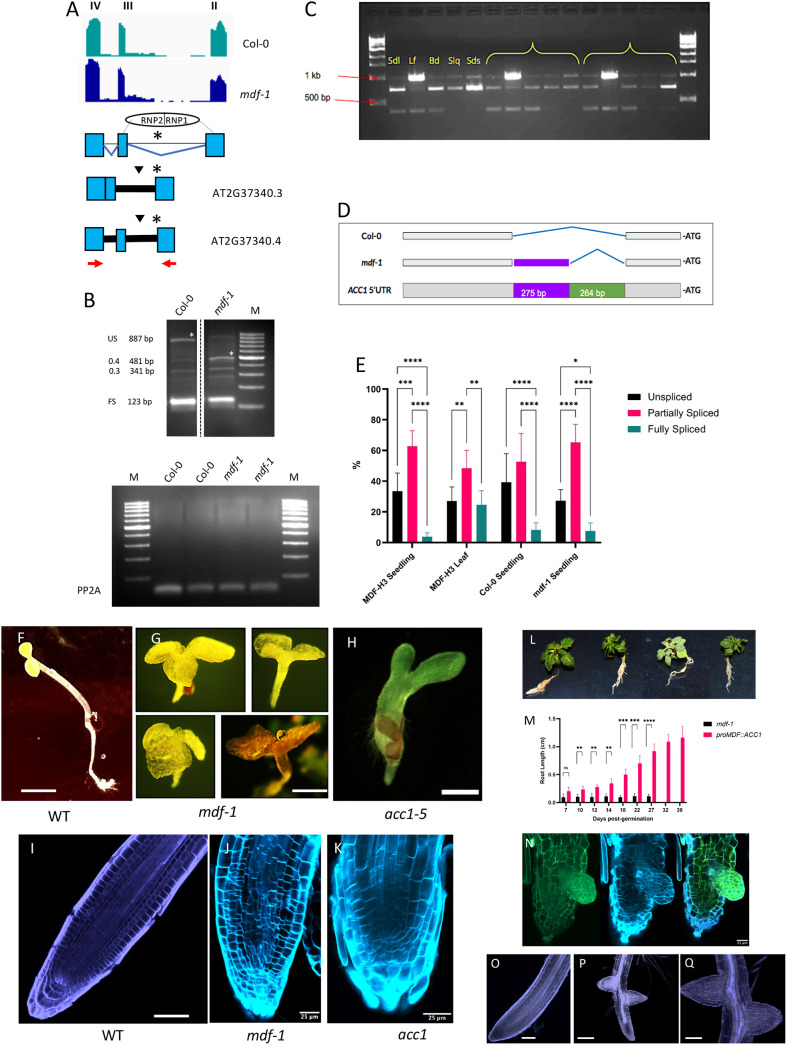
***RSZ33* and *ACC1* transcripts are mis-spliced in *mdf*** **mutants** **and regulate meristem function.** (A) Representation of the *RSZ33* alternative 3′ splice site events. Inverted black triangles indicate premature termination codons. The asterisk and blue lines indicate the position of the alternative 3′ splice site. Red arrows indicate the primer positions used to perform the RT-PCR analysis. (B) Top: Representative root cDNA RT-PCR reactions showing range of splice variants (0.4, AT2G37340.4; 0.3, AT2G37340.3; FS, fully spliced; US, unspliced). Asterisks indicate unspliced variant in Col-0 and significantly increased abundance of isoform AT2G37340.4 of *RSZ33* (481 bp) in *mdf-1* (*P*=0.0147, *n*=3; two-tailed unpaired Student's *t*-test). M, 100 bp size ladder. Bottom: Control RT-PCR with PP2A primers demonstrates equivalent levels of mRNA in two replicates for control and *mdf-1* root samples, using equivalent cDNA concentrations as in top panel. (C) Characterisation of the *ACC1* 5′UTR in 1% agarose gel by RT-PCR. Three size bands were obtained: 1 kb, ∼750 bp and ∼450 bp. The samples were: seedling (Sdl), leaf (Lf), bud (Bd), silique (Slq) and seeds (Sds). Three independent biological replicates were used. (D) Diagrammatic representation of alternative donor site in the *ACC1* 5′UTR in wild type (Col-0) and *mdf-1*. The blue lines indicate the position of the spliced regions. (E) Abundance of *ACC1* 5′UTR splicing variants as determined by RT-PCR and relative abundances on gels determined using Fiji ImageJ software. Asterisks indicate significance levels, using ordinary two-way ANOVA with Tukey's multiple comparison test: *P<0.05, **P<0.01, ***P<0.001, ****P<0.0001; 95% CI. Error bars represent s.e.m., *n*=3. (F) Wild-type seedling at 5 dpg. (G) *mdf-1* seedlings at 27 dpg. (H) *acc1-5* seedling at 5 dpg. (I) Confocal image of wild-type *Arabidopsis* root tip at 7 dpg, stained with propidium iodide. (J) Confocal image of *mdf-1* root tip at 7 dpg, stained with Calcofluor White. (K) Confocal image of *acc1-5* root tip at 7 dpg, stained with Calcofluor White. (L) Transgenic *mdf-1* mutants expressing *proMDF::ACC1:EGFP* at 38 d.p.g., showing phenotypic rescue. (M) Root length comparison between *mdf-1* and *proMDF::ACC1:EGFP* complemented seedlings. Two-way ANOVA and Šídák's post-hoc multiple comparisons test: **P<0.01, ***P<0.001, ****P<0.0001. Bars represent mean+s.e.m., *n*=3. ns, not significant. (N) Confocal image of *proMDF::ACC1:EGFP* seedling root tip at 5 dpg. Left: GFP filter; middle: Calcofluor White filter; right: merged image. In green: GFP; in blue: Calcofluor White. (O) Confocal image of a wild-type root tip at 7 dpg, stained with Calcofluor White. (P,Q) Transgenic *mdf-1* seedlings expressing *proMDF::RSZ33* at 15 dpg, showing partial rescue of primary and lateral root development. Scale bars: 0.5 cm (F,G); 0.25 cm (H); 50 μm (I,Q); 25 μm (J,K,N,O); 100 μm (P).

Sixty-five alternatively spliced gene transcripts were identified associated with the ‘meristem’ GO category (Fig. S3B). One such gene is *RSZ33* (AT2G37340), which encodes an mRNA that is alternatively spliced (Fig. S5A; Palusa et al., 2007). It is strongly expressed in the root meristem (Birnbaum et al., 2003), with four RNA isoforms identified across leaf, root and flower (Simpson et al., 2008), of which three isoforms are found in the root meristem (Li et al., 2020; Fig. 2A,B). *RSZ33* encodes a plant-specific member of the SR protein family, and so potentially is itself a splicing factor. A second gene, *ACC1* (AT1G36160), is required for fatty acid biosynthesis (Baud et al., 2004) and the RNA has three isoforms following splicing in the 5′ UTR in different tissues (Fig. 2C-E). Interestingly, defective expression of each gene (namely in transgenic overexpressers of *RSZ33* and in the *acc1* allelic mutants *gurke* and *pasticcino3*) leads to root growth defects similar to those seen in *mdf*, such as a short root and other embryonic and postembryonic defects (Baud et al., 2004; Kalyna et al., 2003; Fig. 2F-H; Fig. S5B-D). Both *RSZ33* and *ACC1* transcripts are mis-spliced in the *mdf* mutant, producing either premature termination codons that likely lead to NMD of the RNA (Kalyna et al., 2012) and observed reduced transcript levels for *RSZ33* (Fig. 2B) or an alternative 5′ splice donor site usage for *ACC1* (Fig. 2D; Thompson et al., 2023; https://doi.org/10.5061/dryad.b2rbnzskc). These results show that their correct splicing is dependent on MDF activity.

To investigate the functional relationship between MDF, RSZ33 and ACC1, we transformed the *mdf-1* mutant with non-spliceable (i.e. correctly and fully spliced) versions of either *RSZ33* or *ACC1* under the control of the *MDF* promoter, to determine whether either gene could rescue the *mdf* mutant root phenotype. The results show that each introduced gene can partially rescue both primary and lateral root development in the *mdf-1* mutant (Fig. 2I-Q), indicating that both genes are downstream of MDF splicing control and are required for MDF function in root development.

### The splicing factor SR34 is a target of MDF and mediates specific abiotic stress responses

**Fig. 3.**
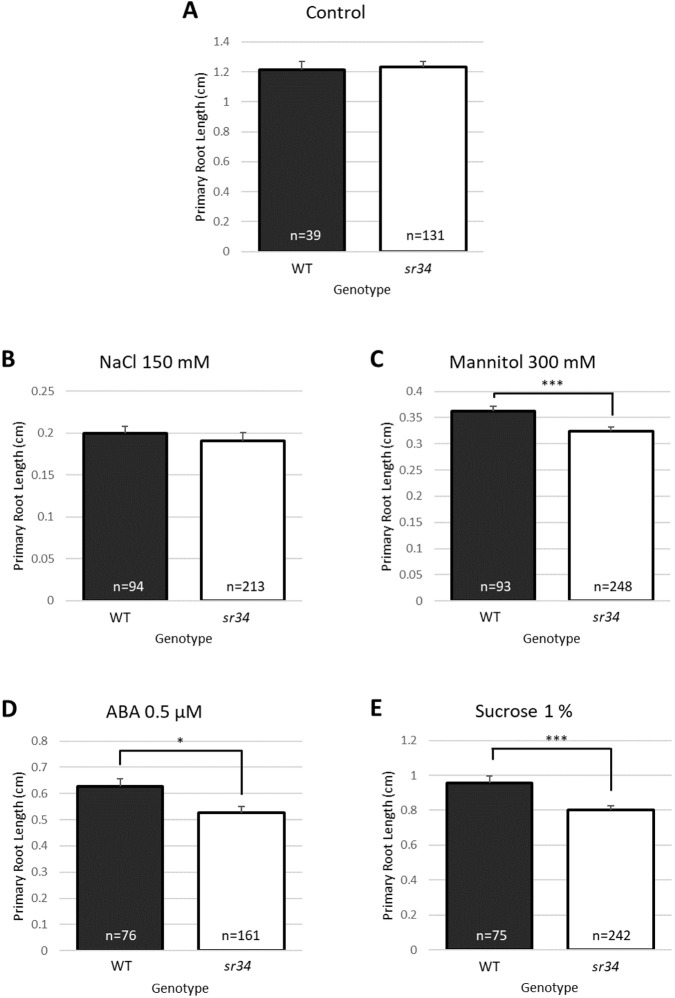
**SR34 mediates specific abiotic stress responses.** (A-E) Primary root lengths were measured for wild-type (WT) and *sr34* mutant seedlings at 7 dpg following growth on half-strength MS medium (Control, A) or half-strength MS medium supplemented with 150 mM NaCl (B), 300 mM mannitol (C), 0.5 μM ABA (D) or 1% sucrose (E). Error bars represent s.e.m., *n*=3. Asterisks indicate significance levels, using ordinary two-way ANOVA with Tukey's multiple comparison test: *P<0.05, ***P<0.001; 95% CI.

SR34 (AT1G02840) is a general splicing factor with at least seven splice isoforms, resulting in protein variants differing in their RS domain (Lopato et al., 1996). SR34 is highly expressed in the root and shoot meristems (Stankovic et al., 2016), and plays an active role in many post-splicing processes, including the export, stability and translation of mRNA. In the *mdf-1* mutant, the *SR34* transcript is mis-spliced compared with wild type, with alternative 3′ splice site selection and a retained intron event (Thompson et al., 2023; https://doi.org/10.5061/dryad.b2rbnzskc). Four *sr34* SALK T-DNA insertion mutants were identified and confirmed as homozygous (Fig. S6), for phenotypic comparison with wild type and *mdf* under standard and abiotic stress conditions. Under control conditions, wild-type and *sr34* seedlings exhibited no discernible difference in primary root length (Fig. 3A; representative allele). A similar result was seen with seedlings grown in the presence of 150 mM NaCl, which induces salinity stress and a shorter primary root compared with controls (Fig. 3B). However, root lengths were significantly differentially reduced when seedlings were grown on media supplemented with 300 mM mannitol and 1% sucrose, which confer osmotic stress, or 0.5 μM ABA, which is a hormone signal activated upon osmotic stress (Rowe et al., 2016), showing that *sr34* mutants are more sensitive than wild type to these specific conditions (Fig. 3C-E). This indicates that MDF plays a role in response to osmotic stress conditions, but not salinity stress, in part via its identified target SR34.

### MDF RNA is spliced and isoforms are regulated by abiotic stress

Given the evidence that MDF promotes stem cell maintenance and represses cell differentiation, we hypothesised that the level of *MDF* expression may provide a mechanism to maintain meristem function under abiotic stress conditions. To test this, we determined whether *MDF* expression or splicing is modulated by environmental stresses that affect root meristem activity and growth.

**Fig. 4.**
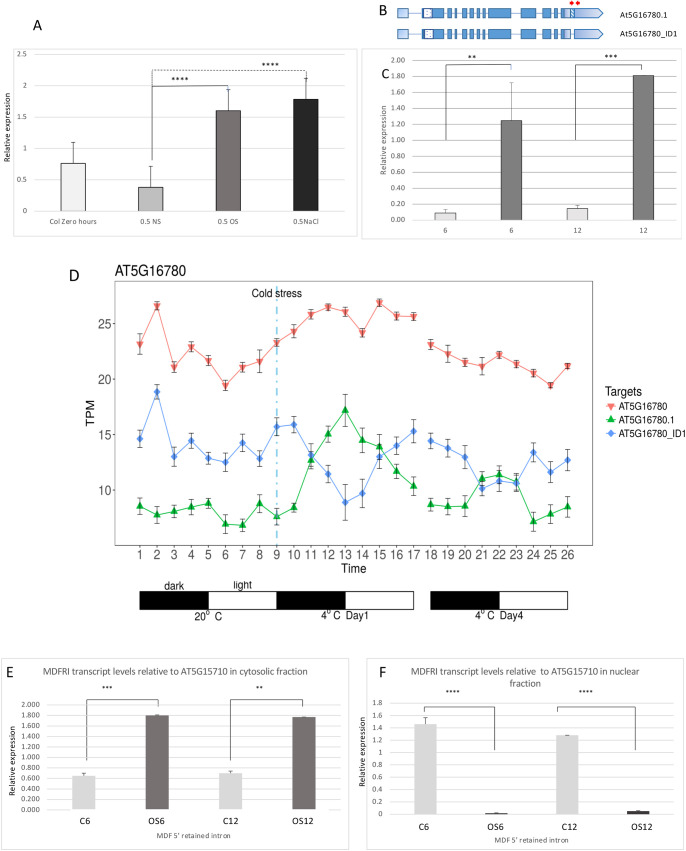
***MDF* expression in response to abiotic stress.** (A) *MDF* expression (measured by RT-qPCR, relative to *UBQ10* expression) in wild-type seedling roots (Col-0) at 7 dpg, at time zero (Col 0 time zero), or transferred for 30 min to standard medium (no stress; NS), 30 min osmotic stress (1.1-1.4 MPa using PEG 8000; OS) or salt stress (150 mM NaCl). Bars represent mean+s.e.m., *n*=3. (B) Gene model of *MDF*. Untranslated regions are shaded pale blue, the C-terminal RS domain is the spotted box, and the 3′ retained intron is shown by the blue diagonal lines. Red arrows represent the primers used in qRT-PCR. (C) qRT-PCR showing the levels of the 3′UTR retained intron in root tissue before and after osmotic stress. Levels significantly rise 6 and 12 h after osmotic shock (1.1-1.4 MPa PEG 80000, dark grey bars). With no stress, the levels remain low and unchanged with time (pale grey bars). Bars represent mean+s.e.m., *n*=3. *x*-axis shows hours after osmotic shock. (D) Switching of *MDF* splicing isoforms (total transcript, AT5G17780; retained intron isoform, AT5G17780.1; spliced isoform, AT5G17780_ID1) under cold stress. At the onset of cold stress (vertical broken blue line), the retained intron transcript (green line) accumulates from a low level within 30 min of treatment, whereas the fully spliced isoform (blue line) declines. Light-dark cycles are indicated. Mined from original data presented by Calixto et al. (2018). Data are mean±s.e.m., *n*=3. *x*-axis shows time after beginning of dark-light cycle, at the temperatures indicated. TPM, transcripts per million. (E,F) RT-qPCR analysis to determine cytosolic (E) and nuclear (F) distribution of MDF isoforms with retained intron under control (non-stress) conditions for 6 or 12 h (C6, C12) or under osmotic stress for 6 or 12 h (OS6, OS12) relative to reference gene *AT5G15710*. Primers MDFUTRintron5′F and MDFRIR were used to detect the unspliced *AT5G16780.1* isoform. Asterisks indicate significance levels, using ordinary two-way ANOVA with Tukey's multiple comparison test: **P<0.01, ***P<0.001, ****P<0.0001; 95% CI. Bars represent mean+s.e.m., *n*=3.

Osmotic stress reduces root growth in *Arabidopsis* via an interacting signalling network involving ABA, ethylene and PIN gene regulation that leads to low accumulated auxin in the root meristem (Rowe et al., 2016). To determine whether MDF may be part of this mechanism, we used RT-qPCR to measure *MDF* expression under osmotic stress (1.1-1.4 MPa using PEG 8000; Rowe et al., 2016) or salt stress. Results show that *MDF* transcription is induced by osmotic and salt stress within 30 min of treatment (*P*<0.01) (Fig. 4A). Interestingly, the *MDF* transcript is itself alternatively spliced, and the levels of a 3′UTR retained intron isoform rose significantly between 6 and 12 h after osmotic shock in root tissue (Fig. 4B,C). This supports a role for MDF in maintaining meristem pathways and suppressing cell death/differentiation under stress conditions. Similarly, under cold stress, we found (using transcriptional data described by Calixto et al., 2018) that there is also isoform switching, with the retained intron transcript accumulating from a low level within 30 min of treatment, whereas the fully spliced isoform declines (Fig. 4D).

Retained introns can influence the intracellular partitioning of transcripts between nucleus and cytoplasm, as a mechanism to avoid NMD (Rausin et al., 2010; Naro et al., 2017), and intron retention is a potential mechanism to ensure transcripts are retained in the nucleus and may be released in response to environmental stress (Jia et al., 2020). To determine whether the retained intron (RI) isoform of the *MDF* transcript is differentially partitioned, we purified nuclei and cytoplasm from *Arabidopsis* roots, isolated RNA and quantified the RI isoform in each fraction by RT-qPCR. The results show that at both 6 h and 12 h after osmotic stress, the RI isoform was relatively abundant in the cytosol (and presumably available for translation to preserve meristem function), whereas in the nucleus its abundance was reduced under osmotic stress, compared with non-stress conditions (Fig. 4E,F). This suggests that there is differential partitioning of the RI isoform of *MDF* between nucleus and cytosol in response to osmotic stress, although further work is required to determine what, if any, biological role the retained intron might play.

Our results show that MDF regulates the growth of roots by controlling the splicing of regulatory mRNAs and the levels of other regulatory gene transcripts. We propose that this represents a network to regulate the balance between stemness and meristem activity on the one hand, and cell division arrest and cell differentiation on the other (Fig. S7). We suggest that this balance is determined in part by the level of MDF, which is itself regulated by stress responses that modulate RNA isoform ratios to regulate growth through the network.

## MATERIALS AND METHODS

### Plant material and growth conditions

T-DNA insertion mutant lines *mdf-1*, *mdf-2*, *acc1* and *sr34* were obtained from the Nottingham *Arabidopsis* Stock Centre (NASC). Seeds were sterilised with 70% ethanol and 10% bleach and then washed five times with sterile distilled water, followed by stratification at 4°C for 4-7 days to synchronise germination and grown on half-strength MS10 medium agar plates as described (Casson et al., 2009).

### RNA extraction and sequencing

RNA was extracted from three independent biological replicates using 7 day post-germination (dpg) seedlings (∼100 mg tissue) grown on half-strength MS10 medium using the Sigma-Aldrich Plant Total RNA Kit (STRN50), with the On-Column DNase I Digestion Set (DNASE10-1SET, Sigma-Aldrich) to eliminate any residual DNA molecules. Tissue was ground in liquid nitrogen before incubation in a lysis solution containing 2-mercaptoethanol at 65°C for 3 min. Solid debris was removed by centrifugation at 14,000 ***g*** for 5 min and column filtration before RNA was captured onto a binding column using the supplied binding solution, which helps to prevent polysaccharide and genomic DNA from clogging the column. Most DNA was removed by wash solutions, and any trace of residual DNA was removed by DNase on the column. Purified RNA was then eluted using RNase-free water.

RNA-Seq from three biological replicate samples was carried out on an Illumina HiSeq 2500 System with the library prepared using the Illumina TruSeq Stranded Total RNA with Ribo-Zero Plant Sample Preparation kit (RS-122-2401). Ribosomal RNA (rRNA) was removed from isolated total RNA using biotinylated, target-specific oligonucleotides on rRNA removal beads. Purified RNA was quality checked using a TapeStation 2200 (Agilent Technologies) with High Sensitivity RNA ScreenTape (5067-5579, Agilent Technologies), and the mRNA was fragmented into 120-200 bp sequences with a median size of 150 bp. Fragmented mRNA was used as a template to synthesise first-strand cDNA using reverse transcriptase and random primers, followed by second-strand cDNA synthesis with DNA Polymerase I and RNase H. Newly synthesised cDNA had a single adenine base added with ligation of adaptors, before being purified and amplified by PCR to make the final library. Library quality control was performed again using a TapeStation with D1000 ScreenTape (5067-5582, Agilent Technologies).

### Pre-processing of RNA-Seq data, differential expression and differential usage analysis

RNA-Seq data were processed and aligned against the TAIR10 (EnsemblePlants) genome using TopHat and indexed with Samtools. Differential expression was determined using DESeq. Alternative splicing analysis was determined using rMATS (Shen et al., 2014; *P*<0.05, a minimum of 10% inclusion difference). Alternative splicing events were visualised using Sashimi plots generated by the Integrative Genomics Viewer (IGV; Robinson et al., 2011).

### Direct mRNA isolation and cDNA preparation for RT-qPCR or RT-PCR

Seedlings were grown until 7 dpg as described above. Roots and cotyledons were separated using a razor blade and frozen immediately in liquid nitrogen. Pools of tissues were used to generate three separate biological samples. Each pool contained approximately 20 mg of root or cotyledon tissue. Total mRNA was extracted using the Dynabeads^®^ mRNA DIRECT™ kit with Oligo(dT)_25_-labelled magnetic beads. Frozen tissue was ground with a sterile plastic micropestle and resuspended in 300 µl lysis buffer. The solution was then forced through a 21-gauge needle in a 1 ml syringe three to five times to shear any DNA and mixed with 50 µl of Dynabeads Oligo(dT)_25_. The kit procedure was followed, with two final washes. To ensure the complete removal of any genomic DNA in the subcellular fractionation experiments, this stage was followed by ezDNase™ treatment in a 10 µl volume (1 µl ezDNase™, 1 µl ezDNase™ 10× buffer and 8 µl sterile H_2_O), 37°C for 2 min, followed by 1 µl DTT and 5 min at 55°C in a heat block.

cDNA was prepared using a SuperScript®IV First-Strand synthesis system directly on the bead solution. For RT-PCR and RT-qPCR, beads were washed in 20 µl 1×SSIV buffer before resuspension in 12 µl sterile H_2_O with 1 µl dNTP 10 mM each mix and incubated for 5 min at 50°C in a Proflex PCR machine (Applied Biosystems). Then, 4 µl 10×SSIV buffer, 1 µl RNase inhibitor and 1 µl Superscript®IV reverse transcriptase were added. The mixture was mixed by pipetting and incubated for 10 min at 50°C, followed by 10 min at 80°C, and then held at 4°C. The 20 µl cDNA mix was stored at −20°C and not eluted from the beads.

Samples were checked for the presence of genomic DNA by PCR using *ACTIN 2* forward and reverse primers. A PCR reaction after 28 cycles with a Tm of 60°C generated a 340 bp product if genomic DNA was a contaminant, 240 bp otherwise. All PCR and sequencing primers are listed in Table S3.

### RT-PCR of splice variants

For each PCR reaction, 0.5-1 µl of cDNA/bead mix were used. RT-PCR was performed with three replicates of *RSZ33* or *ACC1* root-derived cDNA using Phusion™ (Thermo Fisher Scientific) high-fidelity polymerase. PCR products were run on a 1% agarose gel alongside a 100 bp size ladder. Relative levels of *RSZ33* and *ACC1* splice variants were determined using Fiji gel analysis software (Schindelin et al., 2012). Chromatogram peak areas were measured and the ratio results subjected to a two-tailed unpaired Student's *t*-test to determine the significance of any difference. Comparative levels of initial cDNA per replicate were determined relative to PCR-amplified *PP2a* transcript levels.

### Subcellular fractionation

The INTACT line UBQ10::NTF (Marques-Bueno et al., 2016), expressing NTF in the quiescent centre of the root meristem, was supplied by Nottingham *Arabidopsis* Stock Centre (NASC) and used to generate a bulk stock. Seeds were sterilised, stratified and sown on half-strength MS10 medium and harvested at 10 dpg. Roots were cut from the cotyledons using a razor blade, and biological replica pools generated from 20-25 mg of root tissue. Subcellular fractionation was performed based on the method of Hartmann et al. (2018). The final nuclear fraction was resuspended in 200 µl HONDA buffer and, along with 200 µl of cytosolic fraction, mixed with an equal volume of lysis buffer and 50 µl of Dynabeads® labelled with Oligo(dT)_25_. Prior to making the cDNA, the mRNA-bound bead solution was treated with ezDNase™, as described above, to remove genomic DNA.

### PCR with osmotic-shocked root subcellular fractions

Quality control PCR was carried out on 1 µl of cDNA bound to Dynabeads®. To identify genomic DNA contamination, *ACT2* primers were used; a 240 bp band present indicated no contamination. MDF5′UTR and MDFRIR primers amplified the retained intron (120 bp product). Products were run on a 1% agarose gel with a 100 bp size ladder. Phusion™ (Thermo Fisher Scientific) high-fidelity polymerase was used.

### qRT-PCR following osmotic shock

Seedlings at 10 dpg were placed on half-strength MS10 medium agar plates treated to achieve 1.1-1.4 MPa PEG 80000 using the method of Rowe et al (2016). Seedlings were transferred via sterile nylon mesh to reduce any physical shock to the roots. Strips of seedling-bearing mesh were moved from untreated 0.5 MS plates onto osmotic shock and untreated control plates and returned to the growth chamber for 6 and 12 h. A zero-hour set of three biological replicates was harvested to ensure no effect was caused by moving the seedlings. At the determined time, seedlings were harvested, and roots and shoots were separated in the case of the INTACT UBQ10:NTF seedlings. Tissue was transferred to sterile Eppendorf tubes and frozen in liquid nitrogen. mRNA and cDNA were prepared using the Dynabeads™ protocol.

RT-qPCR was conducted using three biological replicates and three technical replicates for each sample. PCR Biosystems PCRBIO™ SYBR Green was used as per the manufacturer's instructions. Relative expression levels were determined using the ΔΔCT method relative to expression of a paired reference gene amplification. The reference gene *AT5G15710* was used owing to its stable expression pattern under osmotic stress and at various developmental stages (Czechowski et al., 2005). Primers are listed in Table S3.

### MDF tertiary structure comparative modelling

A comparative model of MDF was generated by MODELLER (https://salilab.org/modeller/) and superimposed on hSART1 in the human B complex, and the secondary structure prediction for the aligned sequences of MDF1, human hSART1 (5o9z_P) and yeast Snu66 (5NRL_E). MDF template structures were searched using GeneSilico (https://genesilico.pl) and the top-ranked available templates, sections of human hSART1 or yeast Snu66 were used to model MDF. The modelled C-terminal helix of MDF was removed for Fig. S2.

Protein motif similarity modelling was carried out using the KEGG Sequence Similarity Database (SSDB; Sato et al., 2001).

### Yeast two-hybrid analysis

Two *Saccharomyces cerevisiae* strains were used: AH109 (*MATa, trp1–901, leu2–3, 112,ura3–52, his3–200, gal4Δ, gal80Δ, LYS2::GAL1_UAS_-GAL1_TATA_-HIS3, GAL2_UAS_-GAL2_TATA_-ADE2, URA3::MEL1_UAS_-MEL1_TATA_-lacZ*) for bait vectors, and Y187 (*MATα, ura3-52, his3-200, ade2-101, trp1-901, leu2-3, 112, gal4Δ, met–, gal80Δ, URA3::GAL1_UAS_-GAL1_TATA_-lacZ*) for prey vectors. These are described in the Clontech Matchmaker™ GAL4 Two-Hybrid System 3 & Libraries User Manual. Both strains were kindly provided by Professor Patrick Hussey (Durham University, UK). The Frozen-EZ Yeast Transformation II™ Kit (Zymo Research) was used to prepare competent cells using the AH109 and Y187 *S. cerevisiae* strains following the manufacturer's manual.

Synthetic defined (SD) agar with 2% glucose (Formedium) was used for selecting yeast transformants and confirming auxotrophic phenotypes according to the manufacturer's recommendations. Amino acids were added separately in the form of dropout supplements lacking specified nutrients (Complete Supplement Mixture formulations, Formedium). Dropout solutions at 10× concentration were made up using Milli-Q water, filter sterilised, then added to autoclaved SD agar medium (cooled to at least 50°C) to a final concentration of 1× or stored at 4°C. Six types of selection media were prepared, lacking one or more of tryptophan (W), leucine (L), adenine (A) and histidine (H). Liquid SD media was prepared in the same manner, but using media lacking agar.

Competent cells were transformed with 1 µg of each recombinant construct or empty vector into both strains of yeast following the manufacturer's instructions (pGBKT7 constructs into Y187 and pGADT7 constructs into AH109, and also vice versa to test for interaction in the other direction). Cells were combined with the supplied transformation solution (pre-warmed in a water bath to 30°C to improve transformation efficiency) and inverted until homogenous. The transformation mixtures were incubated in a 30°C water bath for 1 h and inverted several times every 15 min, then 200 µl of the transformation mixture was spread onto growth plates. Yeast transformed with pGBKT7 constructs was spread onto –W plates, and pGADT7 constructs onto –L plates, then incubated at 30°C for 2-3 days. Plates with colonies were stored at 4°C for up to 1 month or until needed for mating.

Yeast colonies successfully transformed with bait or prey vectors were used to create resuspensions in liquid SD media with the appropriate –W or –L drop out. Individual colonies (three replicates per genotype) were resuspended in 3 ml of media in 15 ml sterile ventilated tubes and grown overnight at 30°C with agitation at 200 rpm. These resuspensions were then re-streaked onto –W or –L plates to make working plates, and incubated for 2 days at 30°C.

Resuspensions of transformed yeast cells were used to perform matings to generate diploid cells containing two constructs destined to be tested for interaction. On YPDA plates, 5 μl of each yeast type containing the bait construct was spotted and allowed to dry (with three biological replicates per genotype from individual colonies, and three technical replicates from each colony). Five microlitres of each yeast type containing the prey construct was spotted onto the first set of colony drops, and allowed to dry. Plates were incubated for 2 days at 30°C. As negative controls, pGBKT7 constructs were mated against empty pGADT7, and vice versa.

Diploid cells were selected for by resuspending each colony of mated yeast, and spotting 10 μl of each resuspension onto –WL plates. Plates were incubated for 2 days at 30°C. From the interaction assay, resuspensions were made from each resulting colony in 100 μl of sterile Milli-Q water, then transferred to new plates to select for interaction. Resuspended diploid cells were spotted onto –WL plates as controls. For interaction assays, diploid cells were spotted onto –WLA, –WLH with 2.5 mM 3–amino–1,2,4–triazole (3AT; Sigma-Aldrich), and –WLAH with 2.5 mM 3-AT plates. Each spot constituted 10 μl of suspension. Plates were incubated for 5-7 days at 30°C. Haploid cells were also spotted onto each type of media as negative controls.

### BiFC

Fully spliced versions of *MDF*, *BRR2A*, *STA1* (*PRP6*) and *PRP8* were subcloned into Gateway^TM^ vectors pDNOR207 or pDONRZeo and subsequently into both BiFC plasmid vectors pYFN43 and pYFC43 (gift of Dr Patrick Duckney, Durham University, UK) using the Gateway^TM^ Clonase system. Positive clones were identified by colony PCR and then DNA sequenced prior to use in BiFC interactions. Positive controls were pYFC43BIN2 and pYFN43BZR1 (gift of Dr Miguel de Lucas, Durham University, UK).

BiFC constructs (500 µg) were transfected into GV3101 *Agrobacterium tumefaciens* cells and selected with 30 µg/ml gentamycin, 50 µg/ml kanamycin and 50 µg/ml rifampicin. Single colonies were selected and grown then diluted to an OD600 0.2 in LB with 200 µM acetosyringone. GV3101 colonies transformed previously with a p19 construct to suppress gene silencing were also grown. After growth to OD600, 0.4-0.6 cells were resuspended into MMA buffer (MS 5 g/l, MES 1.95 g/l, sucrose 20 g/l, pH adjusted to 5.6 using NaOH) plus 200 µM acetosyringone, and shaken for 1 h at room temperature in the dark. The OD600 determined volumes used combined cultures pYFN*x*+pYFC*x*+p19 in a ratio of 1:1:1 in 1 ml volume.

### Agroinfection and transient expression in tobacco leaf

*Nicotiana benthamiana* plants were grown for 3 weeks at 21°C with 16-h light/8-h dark cycles. Mixed cultures were slowly infiltrated using a 1 ml syringe into the lower leaf epidermal layer until the whole leaf was saturated. Plants were covered and left in the subdued lighting overnight before being returned to the growth cabinet for 3 days, then 0.5 cm sections were taken for microscopic analysis. Imaging of fluorescence in the leaf epidermis was carried out as described previously (Gu et al., 2021).

### Gateway™ cloning

#### BP reaction

From the 5′ upstream region, 1075 bp of the *MDF* promoter and 873 bp of the fully spliced *RSZ33* and *ACC1* coding sequences were synthesised into an artificial fusion within the pEX-A258 vector (Eurofins) with the Gateway™ attB1 and attB2 sequences at the 5′ and 3′ ends. Two sets of primers were designed to amplify the *MDF* promoter and the *pDONR207:: ACC1* backbone from the *pDONR207::ACC1* entry clone (Table S3). The resulting clones were sequenced to ensure no mutations or errors were present. The attB linkers then provided the means to transfer the construct into the Gateway™ entry vector pDONR207 via BP reaction. In a 5 µl reaction, 100 ng of donor vector (pEX-A258-MDFPromRSZ33), 100 ng pDONR207 plasmid, 2 µl of TE buffer pH8 and 1 µl Gateway™ BP Clonase II enzyme (Thermo Fisher Scientific) were combined in a 20 µl PCR tube. After mixing by pipetting, the reaction was placed at 25°C in a heat block overnight. To stop further reaction, 1 µl of proteinase K was added and the reaction tube was heated to 37°C for 10 min, then 2.5 µl of the reaction mix was transformed into Subcloning Efficiency™ DH5α competent cells (Thermo Fisher Scientific) and plated onto L-agar with 50 µg/ml ampicillin selection.

#### Colony PCR

After growth overnight at 37°C, colony PCR was performed to identify positive colonies. Gene-specific PCR primers (Table S3) were used in a reaction with DreamTaq (Thermo Fisher Scientific). Colonies were picked into 20 µl sterile water with a micropipette tip and resuspended. The suspension was also streaked to single colonies on a fresh L-gentamycin 10 µg/ml plate. One microlitre of colony suspension was used per PCR reaction with 1× buffer, 200 µM each dNTP, 0.5 µM of each primer, 1.25 U DreamTaq polymerase in a 50 µl total reaction volume. Reactions were performed at 95°C for 5 min, then 25 cycles of 95°C for 30 s, 56°C for 30 s, 72°C for 30 s, followed by one cycle of 5 min at 72°C. For controls, 1 ng of pEX-A258-MDFPromRSZ33/ACC1 was used. PCR products were analysed on a 1% agarose gel with 100 bp markers.

#### DNA sequencing

PCR-positive clones were isolated and each cultured to prepare plasmid DNA for sequencing. Construct fidelity was confirmed by the Sequencing Service, Department of Biosciences, Durham University, UK. DNA sequences were analysed using SnapGene software.

#### LR reaction and GV3101 transformation

One-hundred nanograms of the vector pMDC107_B with Basta resistance gene (gift of Dr J. Kroon, Durham University, UK) was used as the destination vector for the Gateway™ LR reaction containing 100 ng of pDONR207MDFprom::RSZ33, 3 µl TE pH 8 buffer and 1 µl Gateway™ LR Clonase II enzyme (Thermo Fisher Scientific). The reaction was mixed and incubated at 22°C for 3 h, followed by the addition of 1 µl proteinase K and a further incubation at 37°C for 10 min. The whole reaction was used to transform MAX Efficiency™ DH5α competent cells (Thermo Fisher Scientific) and selected on L-agar+kanamycin 50 µg/ml. To produce the *proMDF::ACC1:EGFP* expression clone, purified *proMDF::ACC1* entry clone was used for the LR reaction, cloned into the pJK1243 destination vector, and introduced into chemically competent DH5α cells. Positive colonies were confirmed by PCR. Sequencing of the expression clone was performed only to ensure that the protein fusion between *ACC1* and *GFP* DNA sequences was in the correct reading frame.

Plasmid DNA was prepared from a positive clone, and 1 µg was used to transform the *Agrobacterium tumefaciens* strain GV3101 (lab stocks); a negative H_2_O control was included. Positive transformants were selected on gentamycin 30 µg/ml, kanamycin 50 µg/ml and rifampicin 50 µg/ml. *Arabidopsis* was transformed with the respective *proMDF::RSZ33* and *proMDF::ACC1:EGFP* constructs by the floral dip method (Clough and Bent, 1998).

#### *mdf-1* complementation by transformation

To determine whether non-spliceable versions of *RSZ33* or *ACC1* could rescue the *mdf-1* mutant, heterozygous *mdf-1* mutants were used to generate flowering plants for transformation, as homozygote mutants do not flower. Six plants per dipping were transformed by floral dip (Clough and Bent, 1998) using *Agrobacterium* containing either *proMDF::RSZ33* or *proMDF::ACC1:GFP* (fully spliced variants). Seeds from several individual transformation events were collected after 3-5 weeks, when the siliques were brown and dry, for selfing to generate mutants homozygous for *mdf-1* and containing either the *proMDF::RSZ33* or *proMDF::ACC1:GFP* transgenes, for phenotypic analysis.

#### Selection of transformants

Stratified T1 seeds (2 days at 4°C) from independent plants were sown at high density on compost and grown at 22°C (∼3000 lux) in a growth chamber (Sanyo Electric) in a 16 h photoperiod. After 10 days, putative *proMDF::RSZ33*×*mdf-1* seedlings were sprayed three times with 250 mg/l commercial Basta (Kurtail Evo) at 2 day intervals (Harrison et al., 2006). Surviving transformants were transferred to individual pots and allowed to flower. T2 seeds were collected in a covering seed bag to prevent cross-contamination. Once brown and dry, the siliques were harvested and stored.

Transformation was confirmed by genotyping. Cotyledons at 10 dpg were used in two PCR reactions using the MyTaq™ Plant-PCR kit (Meridian Biosciences®). Slices of cotyledon (2×2 mm) were added directly to a 50 µl reaction. Two separate PCR reactions were performed to identify the construct with relevant primers to determine whether the *MDF* promoter is fused to the *RSZ33* or *ACC1* coding sequences.

To identify any developmental effect of the *MDF* promoter coupled to non-spliceable versions of *RSZ33* or *ACC1* on the *mdf-1* phenotype, 10 dpg seedlings were selected. *proMDF::RSZ33*×*mdf-1* seeds were sown onto medium containing 50 mM phosphinothricin solution (Basta active ingredient; Sigma-Aldrich) in 0.1% agar (Harrison et al., 2006). *proMDF::ACC1:GFP*×*mdf-1* homozygous mutant plants were selected by phenotype, GFP fluorescence and PCR. Seedlings with the typical *mdf-1* phenotype were transferred to a new plate to be observed under a fluorescence stereo microscope (Leica M165 FC Fluorescence stereo microscope equipped with a Leica DFC 420C camera) before confocal imaging.

#### Root tissue preparation and microscopy

Separated roots were individually placed in a sterile plastic well plate and then fixed in 4% paraformaldehyde for 30 min under vacuum. Roots were then gently washed twice in 1× PBS. Samples were stored in ClearSee solution (Kurihara et al., 2015) for a minimum of 4 days prior to observation by microscopy. Control wild-type root tissue was prepared for comparison. For seedlings stained with Calcofluor White, 0.1% Calcofluor White in ClearSee solution was prepared and the seedlings were stained for 30 min with gentle shaking. Seedlings were washed in ClearSee for 30 min prior to imaging. Imaging was carried out using a Zeiss 800 laser-scanning confocal microscope 20× objective with excitation at 405 nm.

## Supplementary Material

10.1242/develop.201476_sup1 Supplementary information
