# Supplementary material for: MERISTEM-DEFECTIVE regulates the balance between stemness and differentiation in the root meristem through RNA splicing control
Source: Development. 2023 Apr 5;150(7):dev201476. doi: 10.1242/dev.201476 (PMC10112893; doi:10.1242/dev.201476)
Supplement: Supplementary information [file develop-150-201476-s1.pdf]

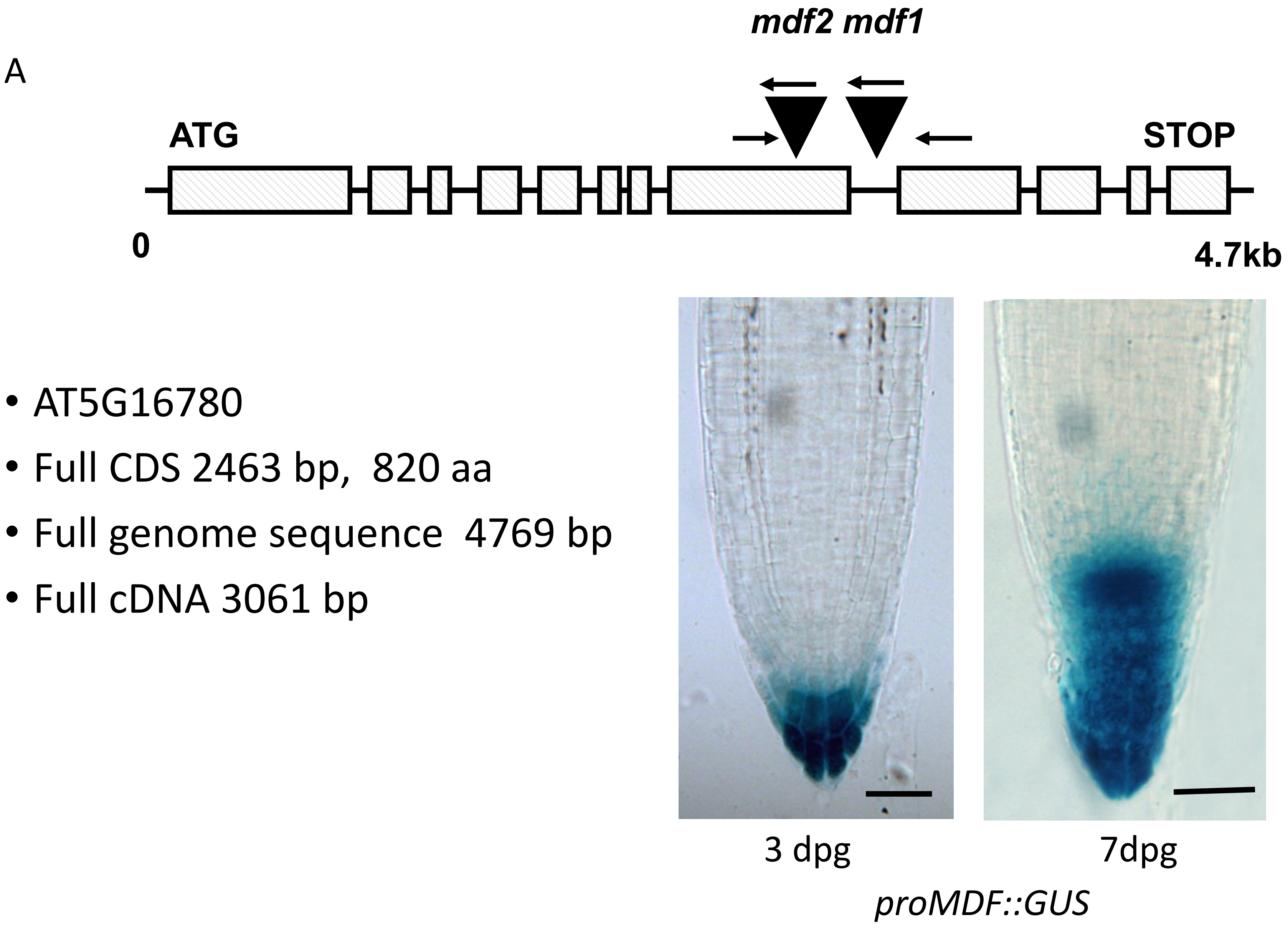

Fig. S1. The *MDF* gene and protein

A. Organization of the *MDF* gene (AT5G16780) showing sites of mutations (*mdf-1*, *mdf-2* - black triangles) and expression of the proMDF::GUS gene fusion in the root tips of transgenic Arabidopsis at 3 and 7 dpg.

Development • Supplementary information

SSDB Motif Search Result

Organism : T00041
Gene : AT5G16780
Definition : U4/U6.U5 tri-snRNP-associated protein 1

Motif id	From	To	Definition	E value	Score
ps:ARG_RICH	9	130	Arginine-rich region profile.	-	46
pf:SART-1	131	727	SART-1 family	1.6e-116	-
ps:NLS_BP	296	310	Bipartite nuclear localization signal profile.	-	4

Search GENES with the same motifs

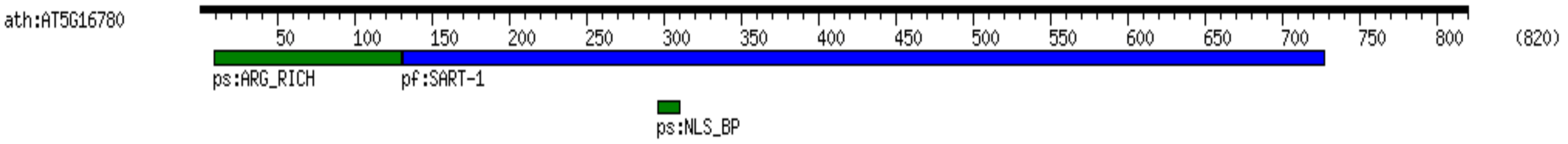

Fig. S1B. SSDB protein motif search for MDF, showing strong similarity to SART1 family proteins.

Development • Supplementary information

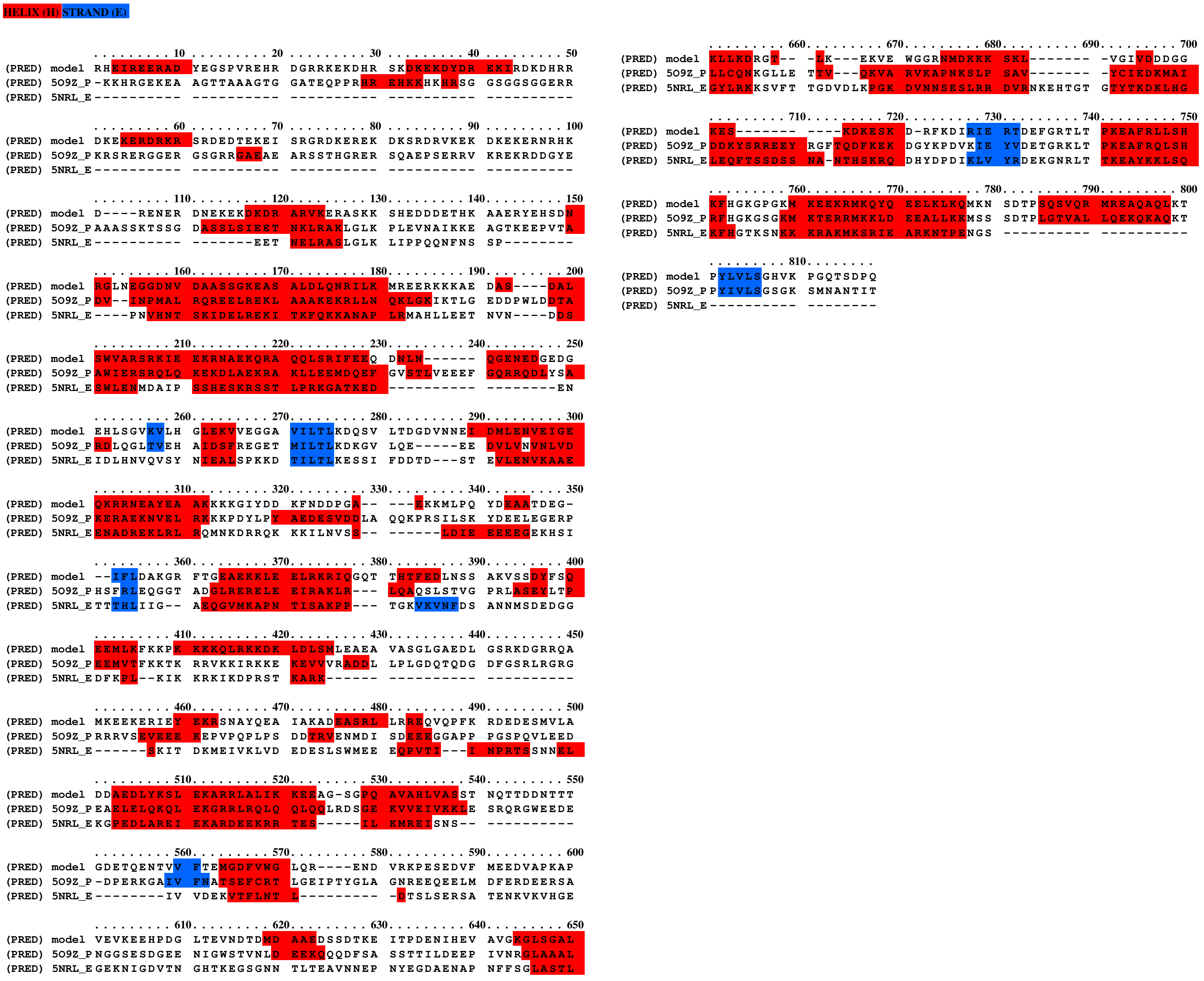

Fig. S1C. Predicted helical (red) and strand (blue) regions in MDF (model), hSART-1 (509Z_P) and SnU66 (5NRL_E) proteins.

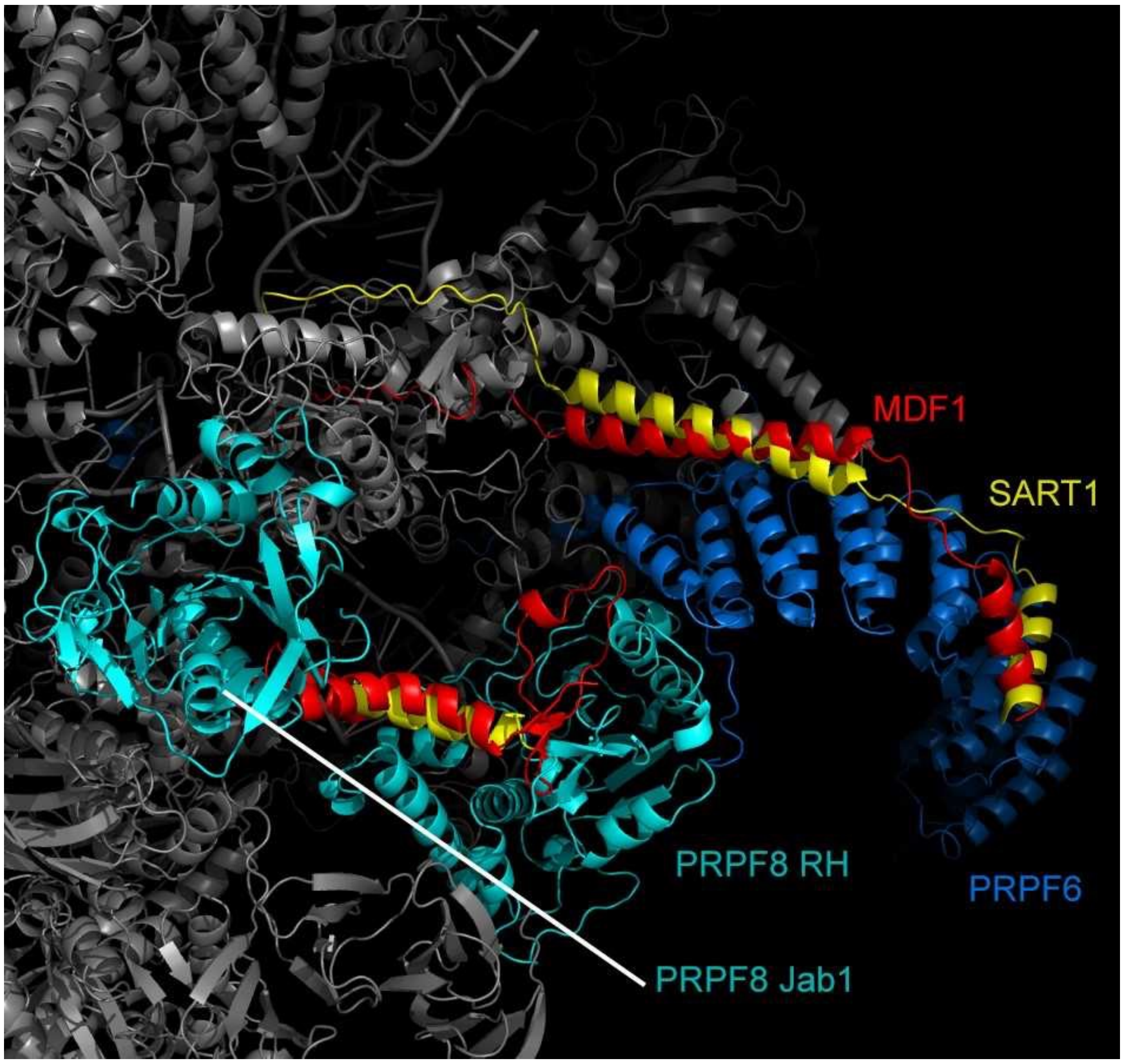

Fig. S2. Predicted MDF structure and protein interactions in the plant spliceosome.
A. Comparative model of the putative 3-dimensional structure of the MDF/DOT2 protein (red) suggests a strong structural similarity with hSART-1 (yellow). Predicted relationships with PRPF6 and PRPF8 (cyan) is shown.

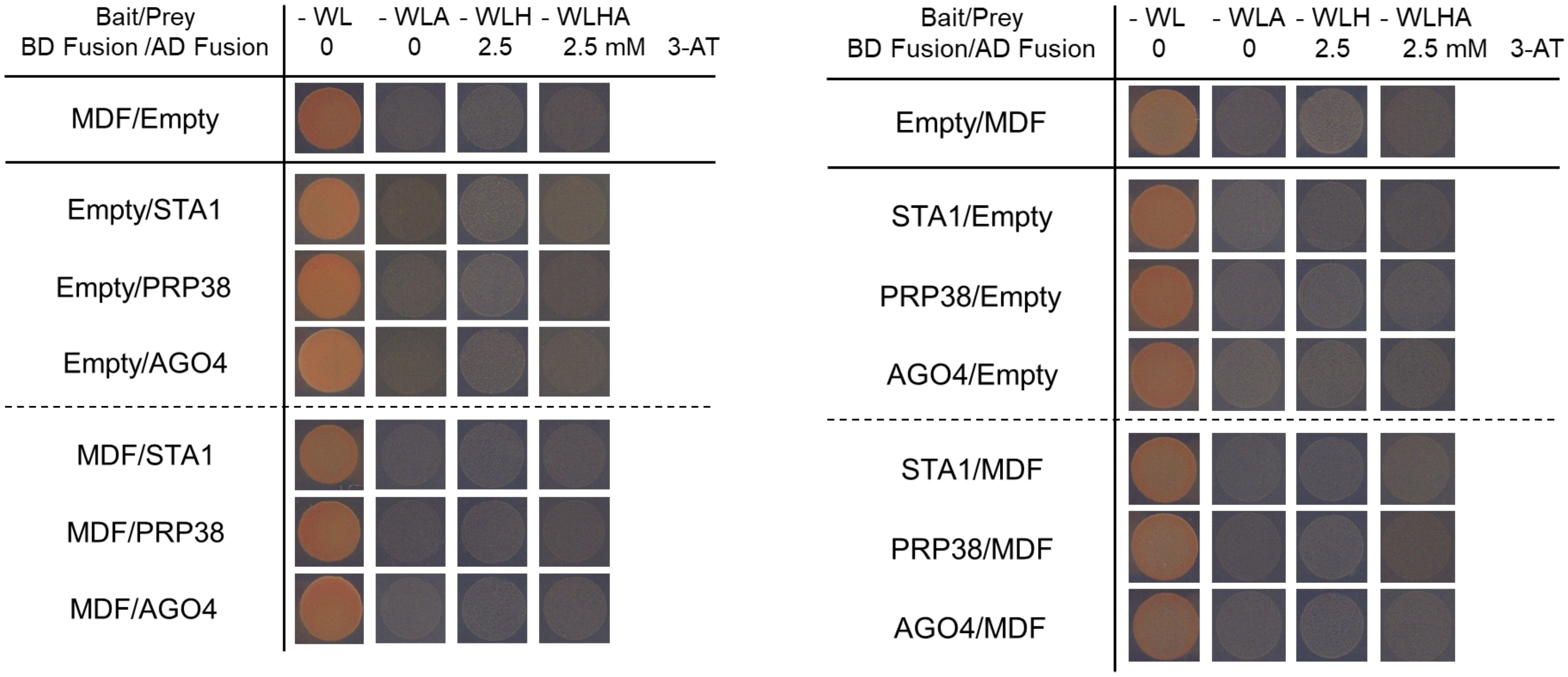

Fig. S2B. Yeast two-hybrid one-on-one tests.

Left panel: Full length MDF-BD fusion protein in pGBKT7 was mated against full length STA1-, PRP38- and AGO4-AD fusion proteins in pGADT7. Each recombinant construct was mated against empty vectors as negative controls. Three colonies of each yeast type were spotted in triplicate on to different selection media lacking a combination of either tryptophan (W), leucine (L), adenine (A), and/or histidine (H). One representative spot from the biological and technical replicates of each yeast strain on each type of media was chosen for this figure.

Right panel: Full length STA1-, PRP38- and AGO4-BD fusion proteins in pGBKT7 were mated against full length MDF-AD fusion protein in pGADT7, to test for interactions in the opposite conformation. Each recombinant construct was mated against empty vectors as negative controls. Three colonies of each yeast type were spotted in triplicate on to different selection media to select for protein interaction at different levels of stringency. For this figure, one representative spot was chosen from the biological and technical replicates of each yeast strain on each type of media.

Development • Supplementary information

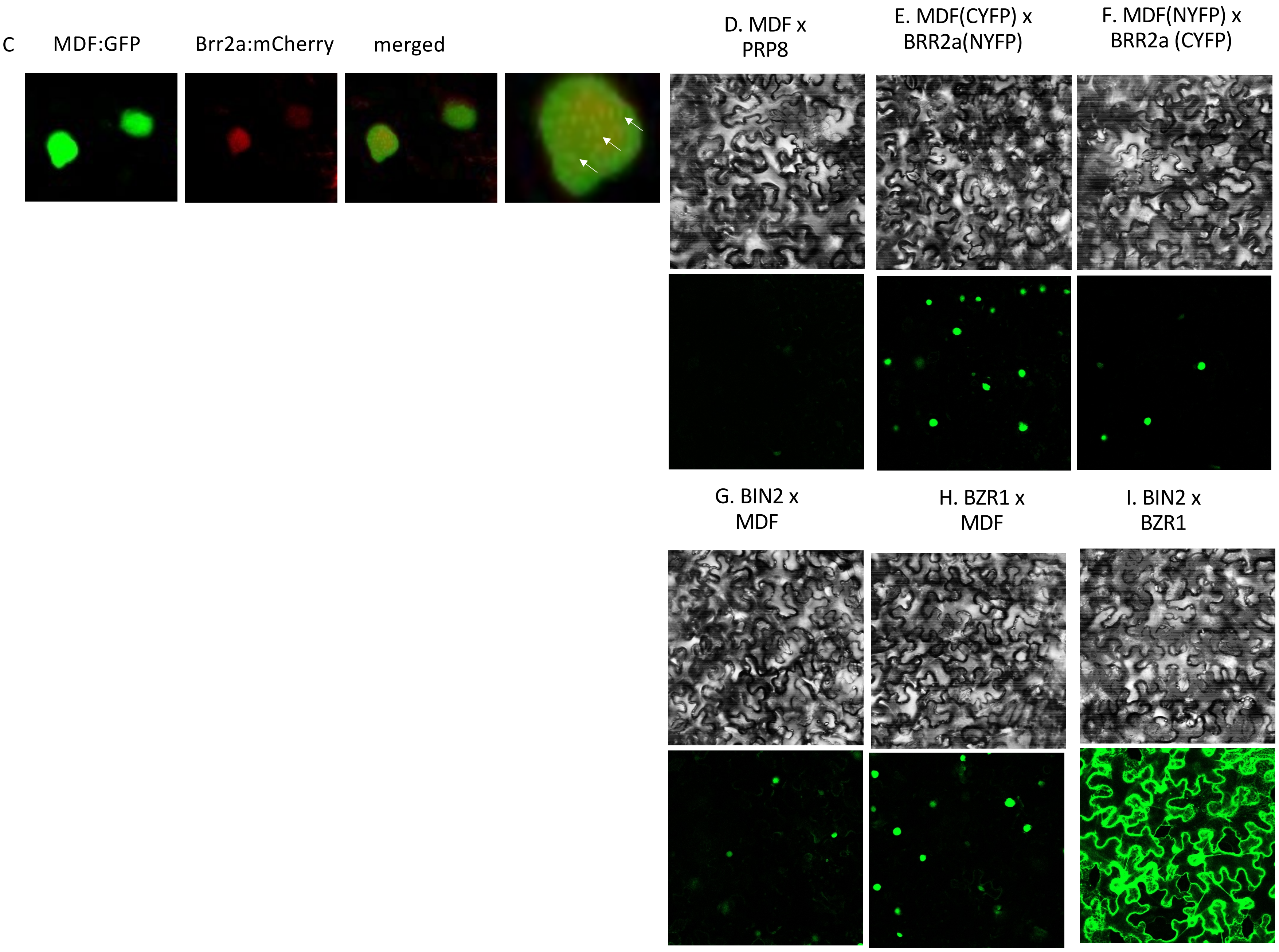

Fig. S2C-I. C. Co-localization between MDF:GFP and Brr2a:mCherry in the nucleus; arrows show co-localization in nuclear speckles. D-I Bimolecular fluorescence complementation (BiFC) following transient gene expression in *Nicotiana bethamiana* leaves shows no interaction between MDF and PRP8 (D), but interaction between MDF and BRR2a (E, F) MDF and BIN2 (G), and MDF and BZR1 (HG). BIN2 and BZR1 interaction acts as a positive control (I).

Development • Supplementary information

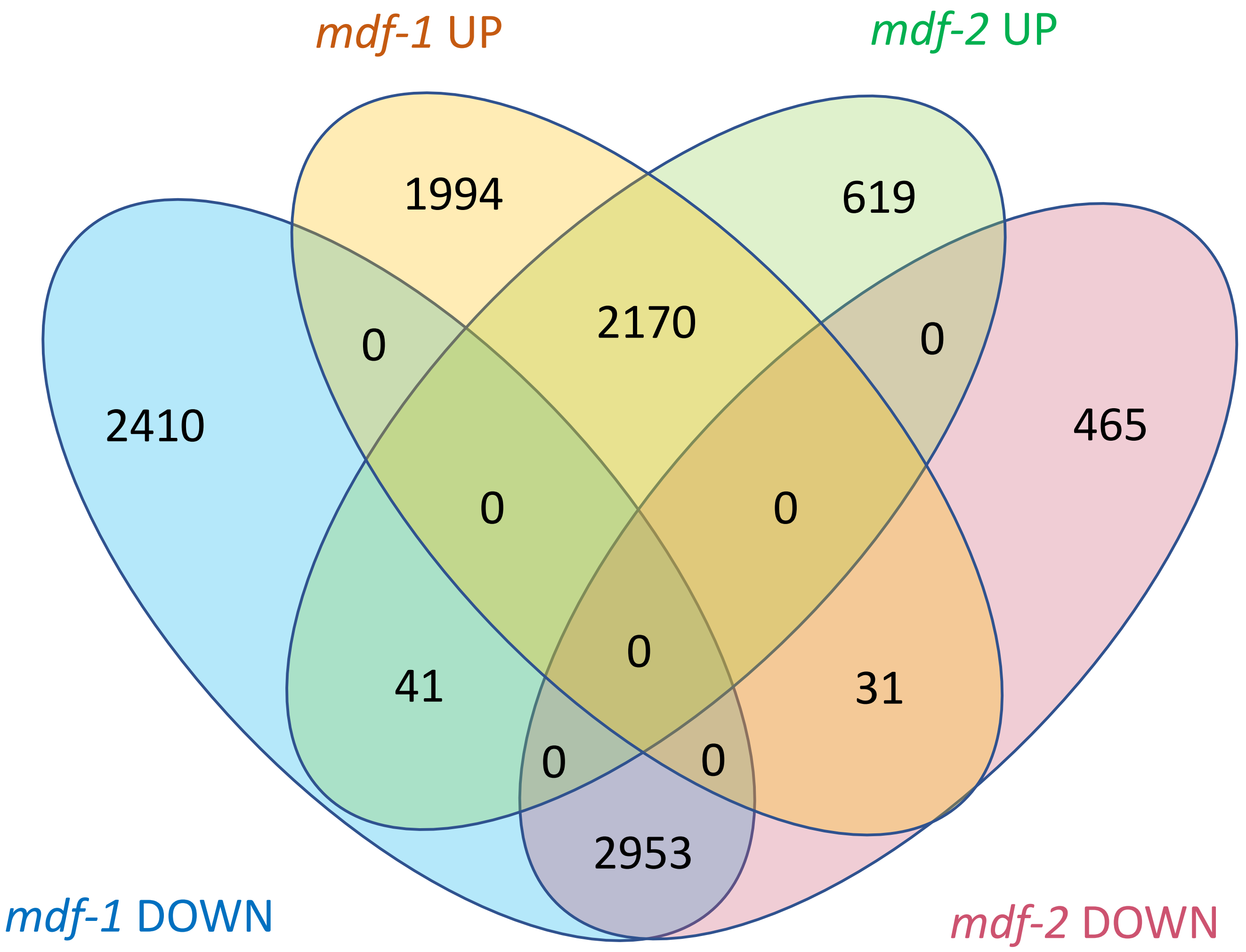

Fig. S3. Transcriptional analysis of *mdf* mutants

A. Venn diagram showing the number of differentially expressed genes (DEGs) in *mdf-1* and *mdf-2* homozygous transgenic 7 d.p.g. seedlings following RNA-Seq, with adjusted p value < 0.05, and log2 fold change (log2fc) >1. Each oval contains all up- or down-regulated genes in one of the genotypes, and the overlapping parts represent numbers of genes meeting the conditions of more than one encircling oval. Percentage under each number is calculated by dividing each number by the total number of DEGs in the diagram.

Development • Supplementary information

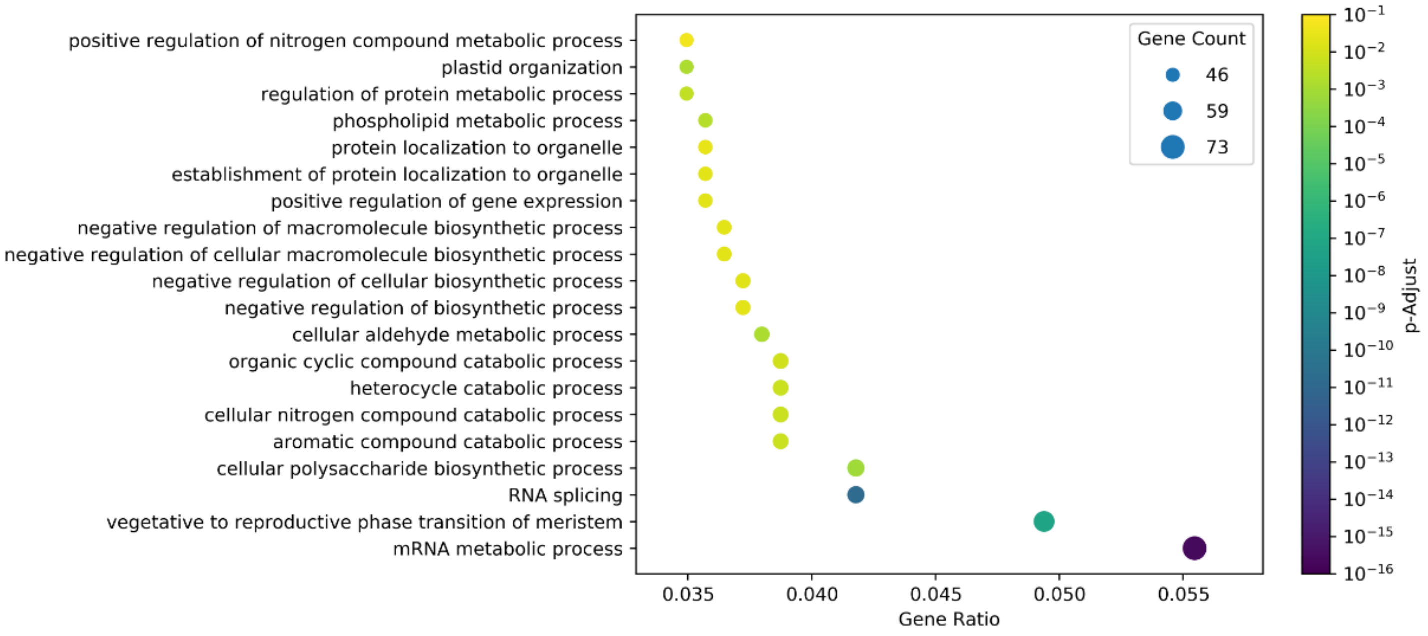

Fig. S3B. Gene enrichment analysis dotplot of the 2015 alternative splicing events identified by rMATS analysis with a p-value < 0.01 and minimum ± 10 % inclusion difference. The most represented GO term, the largest dot, contained mRNA metabolic process candidate genes followed by vegetative to reproductive phase transition of meristem.

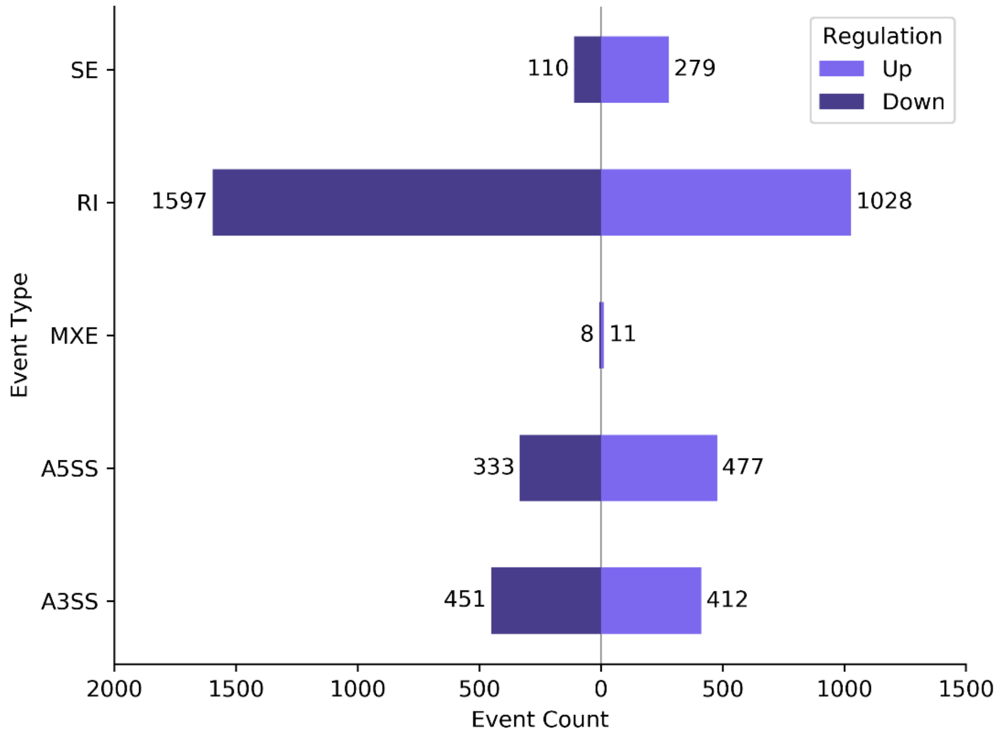

Fig. S3C. Comparison of the alternative splicing events between Col-0 and *mdf-1,* identified by rMATS analysis of the RNAseq data. The events compared were SE (skipped exon), RI (retained intron), MXE (multiple exon events), A5SS (alternative 5'splice sites) and A3SS (alternative 3'splice sites).

Treemap of Enriched GO Terms in Significantly Upregulated Genes from *mdf-1* Mutant Seedlings

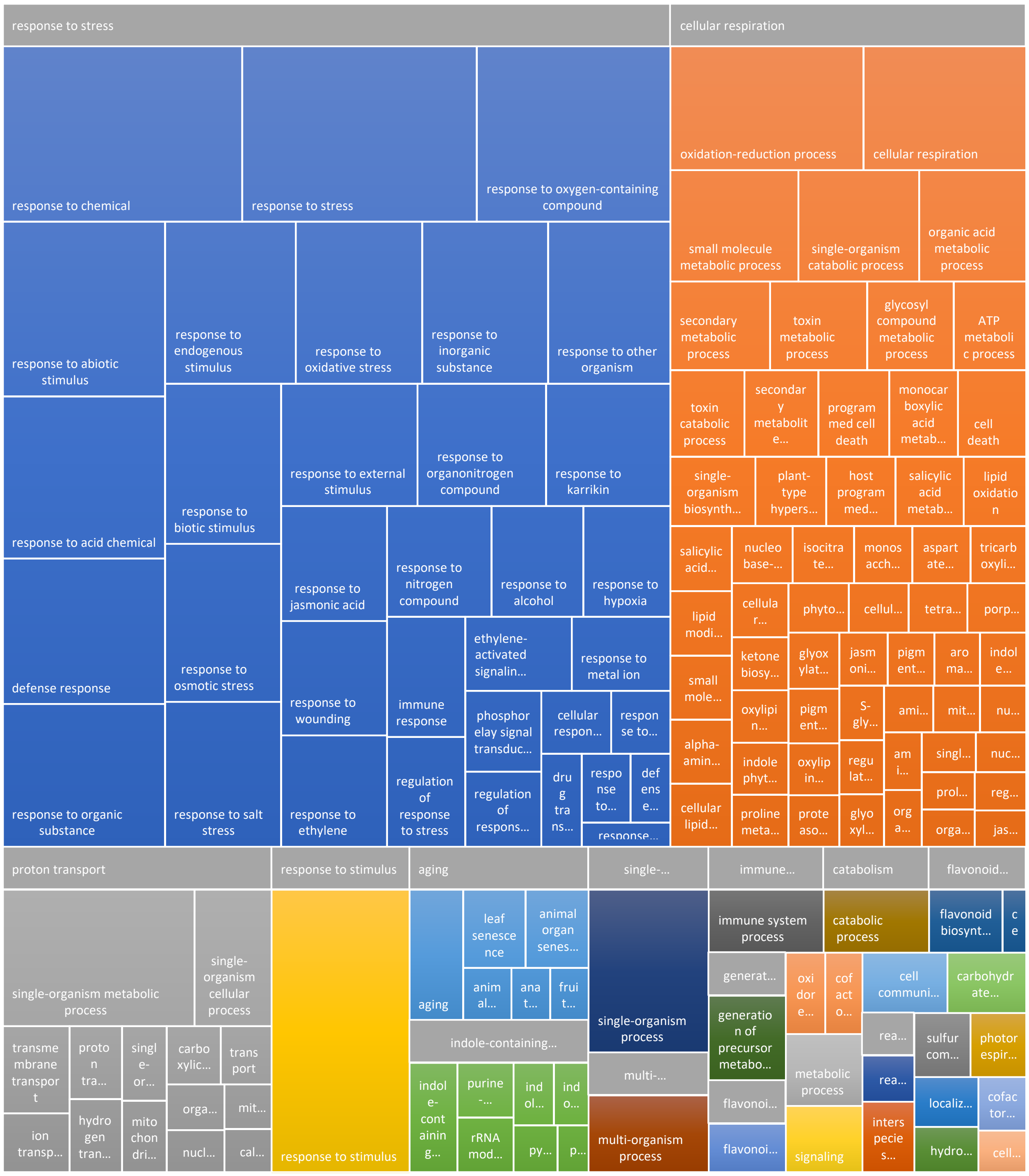

Fig. S3D. Tree map output from REVIGO showing the clustering of enriched gene ontology (GO) terms in the significantly upregulated genes in *mdf-1* mutant RNA sequencing data, with adjusted P value <0.05 and $\log_2$ fold change ($\log_2$fc) >1. Each coloured rectangle represents a GO term cluster, and each colour with its grey bar on top and colour key below the tree map shows a supercluster of related clusters. The colour key is ordered by total size of the cluster from left to right on each row, then top down across. The size of each coloured rectangle represents the absolute value of the $\log_{10}$ P-value ($|\log_{10}$ q-value$|$) of its cluster.

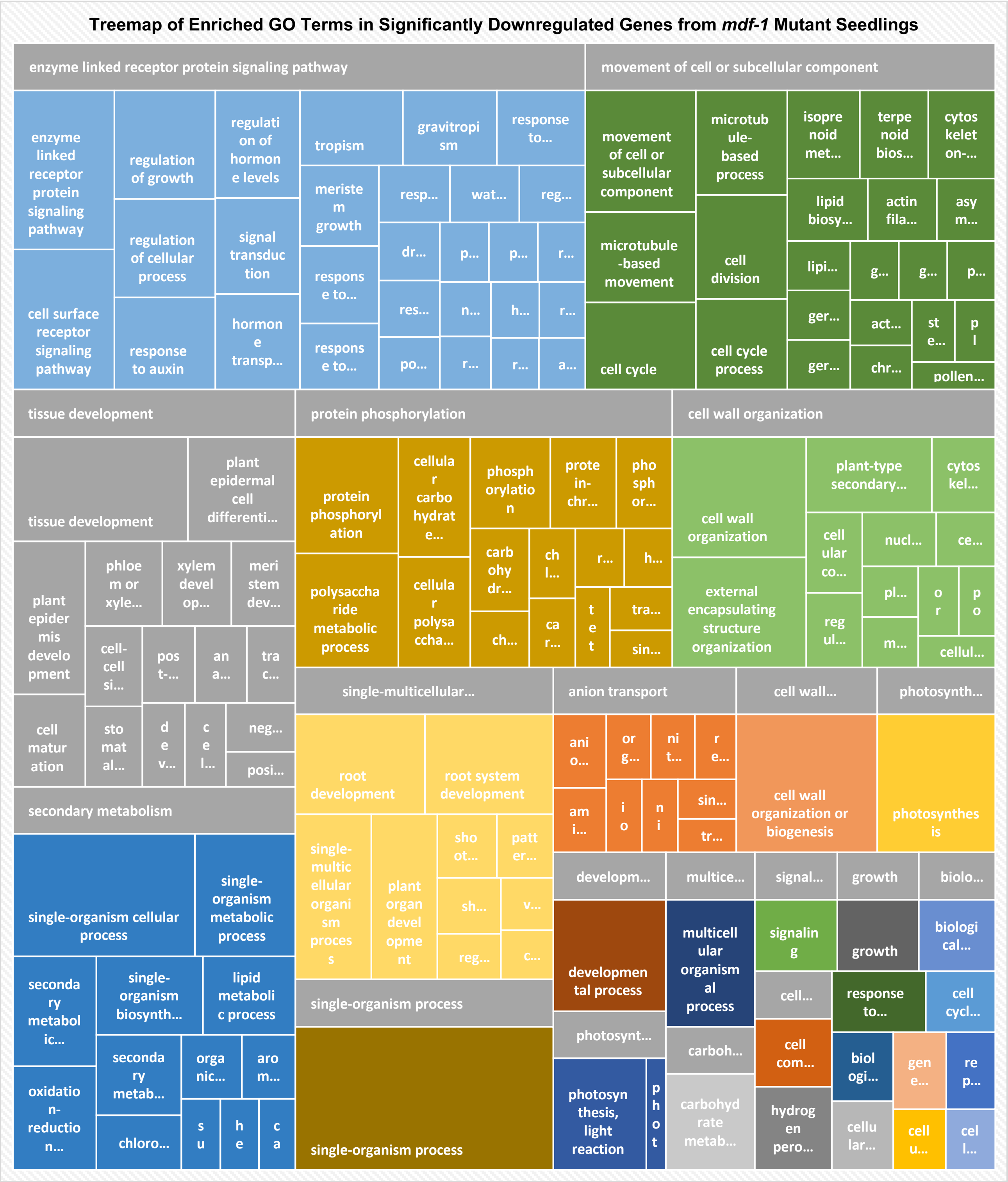

Fig. S3E. Tree map output from REVIGO showing the clustering of enriched gene ontology (GO) terms in the significantly downregulated genes in *mdf-1* mutant RNA sequencing data, with adjusted P value <0.05 and $\log_2$ fold change ($\log_2$fc) <1. Each coloured rectangle represents a GO term cluster, and each colour with its grey bar on top and colour key below the tree map shows a supercluster of related clusters. The colour key is ordered by total size of the cluster from left to right on each row, then top down across. The size of each coloured rectangle represents the absolute value of the $\log_{10}$ P-value (|$\log_{10}$ q-value|) of its cluster.

Development • Supplementary information

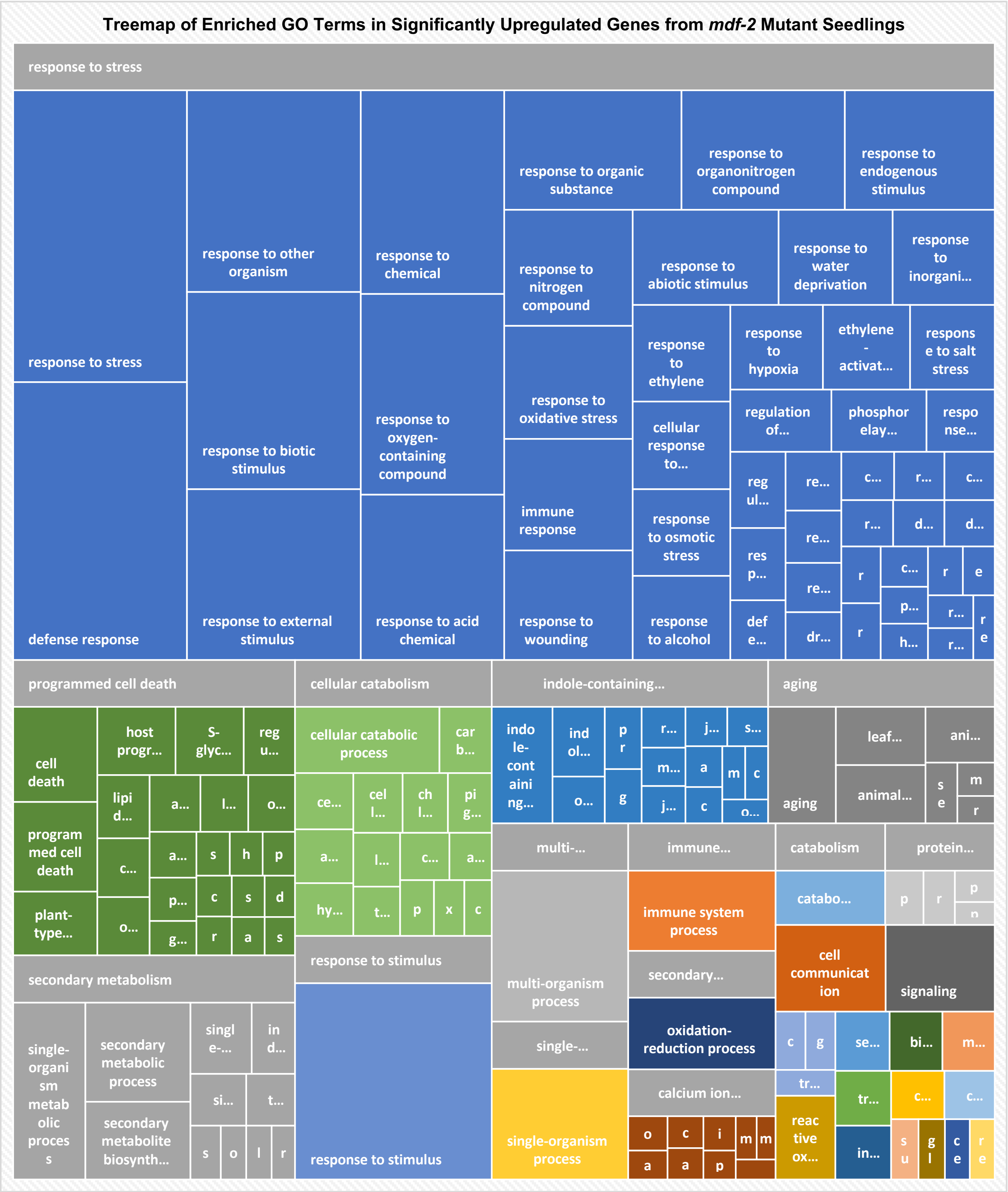

Fig. S3F. Tree map output from REVIGO showing the clustering of enriched gene ontology (GO) terms in the significantly upregulated genes in *mdf-2* mutant RNA sequencing data, with adjusted P value <0.05 and $\log_2$ fold change ($\log_2$fc) >1. Each coloured rectangle represents a GO term cluster, and each colour with its grey bar on top and colour key below the tree map shows a supercluster of related clusters. The colour key is ordered by total size of the cluster from left to right on each row, then top down across. The size of each coloured rectangle represents the absolute value of the $\log_{10}$ P-value ($|\log_{10}$ q-value$|$) of its cluster.

Treemap of Enriched GO Terms in Significantly Downregulated Genes from *mdf-2* Mutant Seedlings

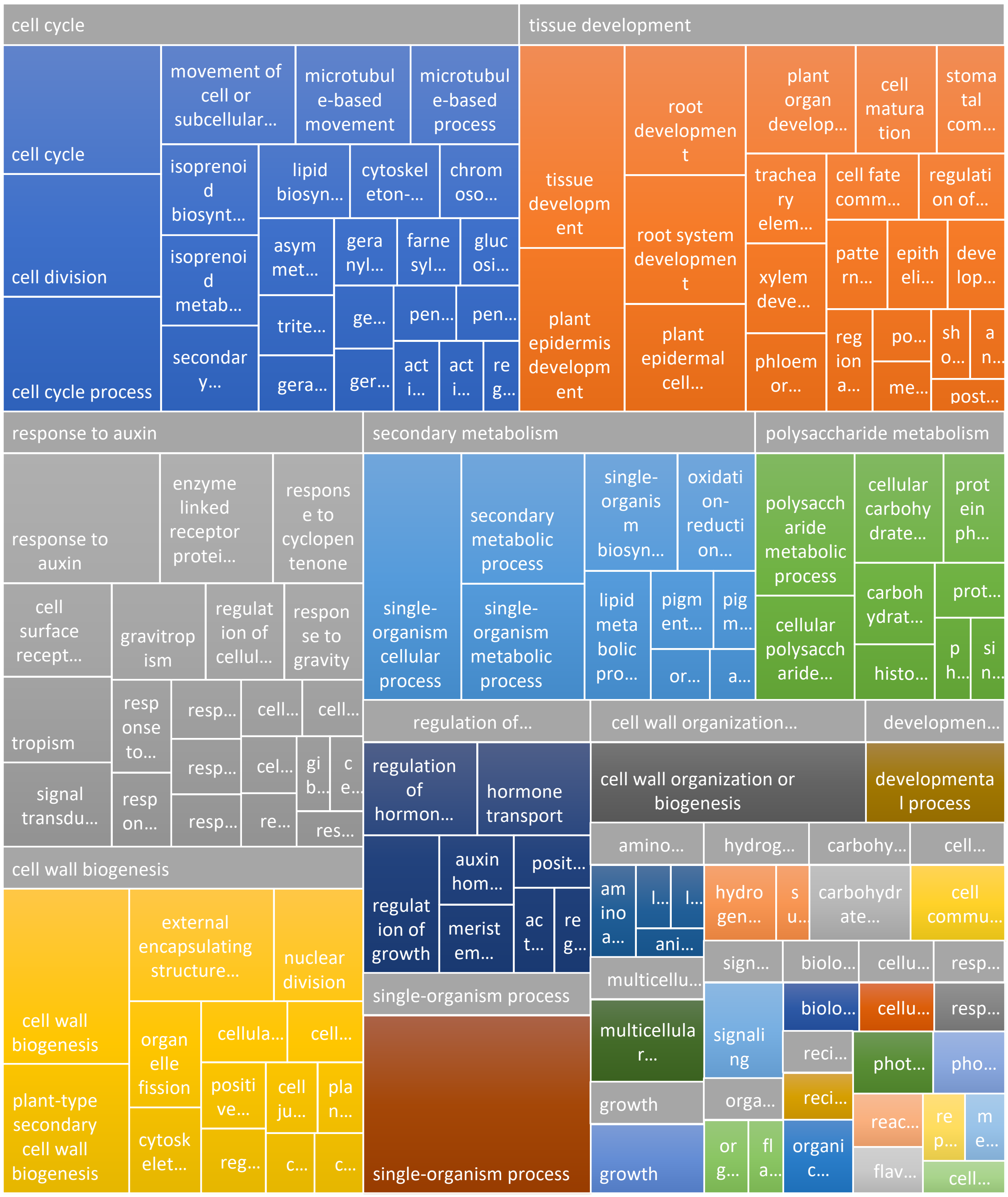

Fig. S3G. Tree map output from REVIGO showing the clustering of enriched gene ontology (GO) terms in the significantly downregulated genes in *mdf-2* mutant RNA sequencing data, with adjusted P value <0.05 and $\log_2$ fold change ($\log_2$fc) <1. Each coloured rectangle represents a GO term cluster, and each colour with its grey bar on top and colour key below the tree map shows a supercluster of related clusters. The colour key is ordered by total size of the cluster from left to right on each row, then top down across. The size of each coloured rectangle represents the absolute value of the $\log_{10}$ P-value ($|\log_{10}$ q-value$|$) of its cluster.

Development • Supplementary information

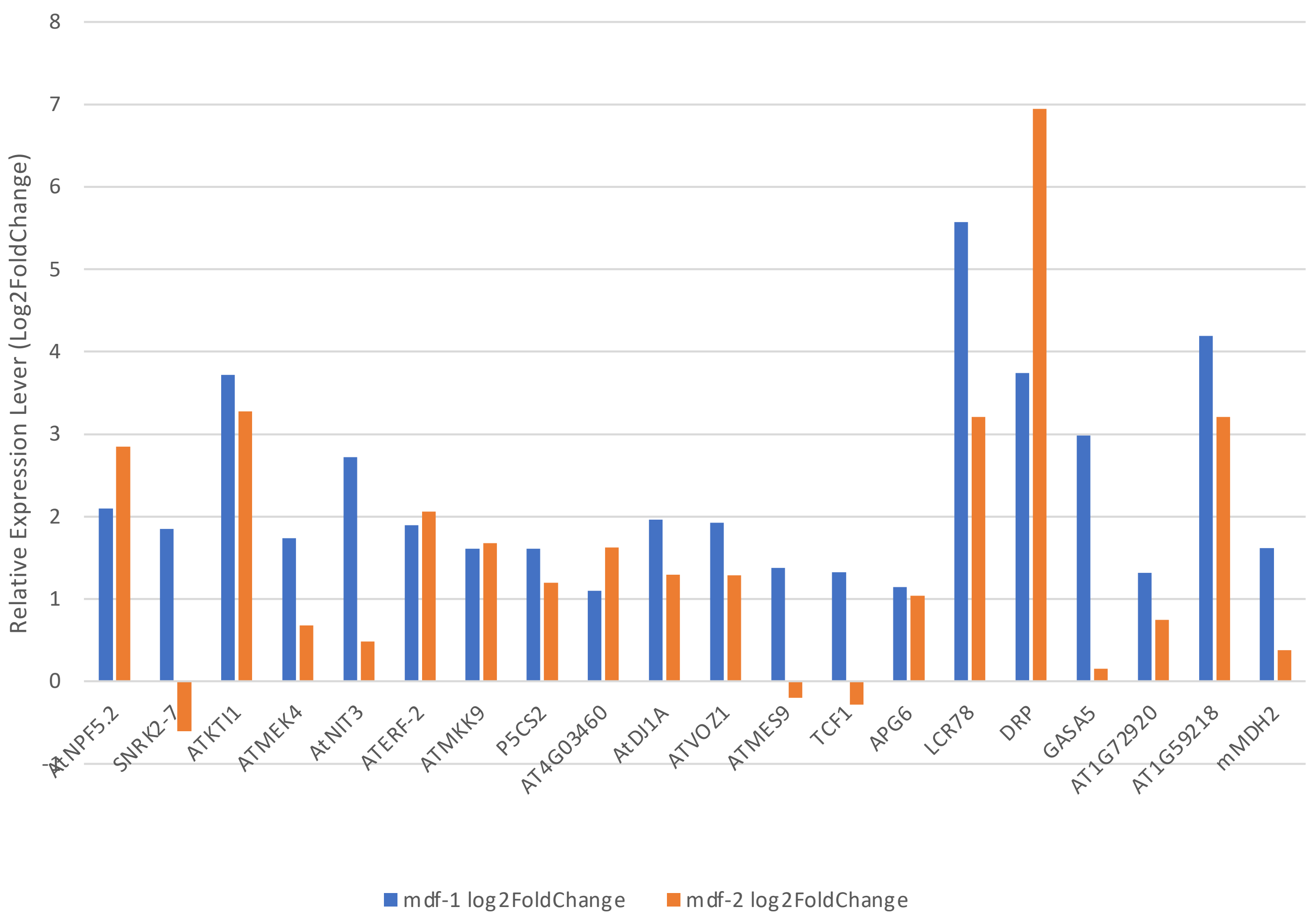

Fig. S3H. Differential expression analysis (DeSeq2) of stress response genes (as revealed as GO terms) in *mdf-1* and *mdf-2* compared to Col-0.

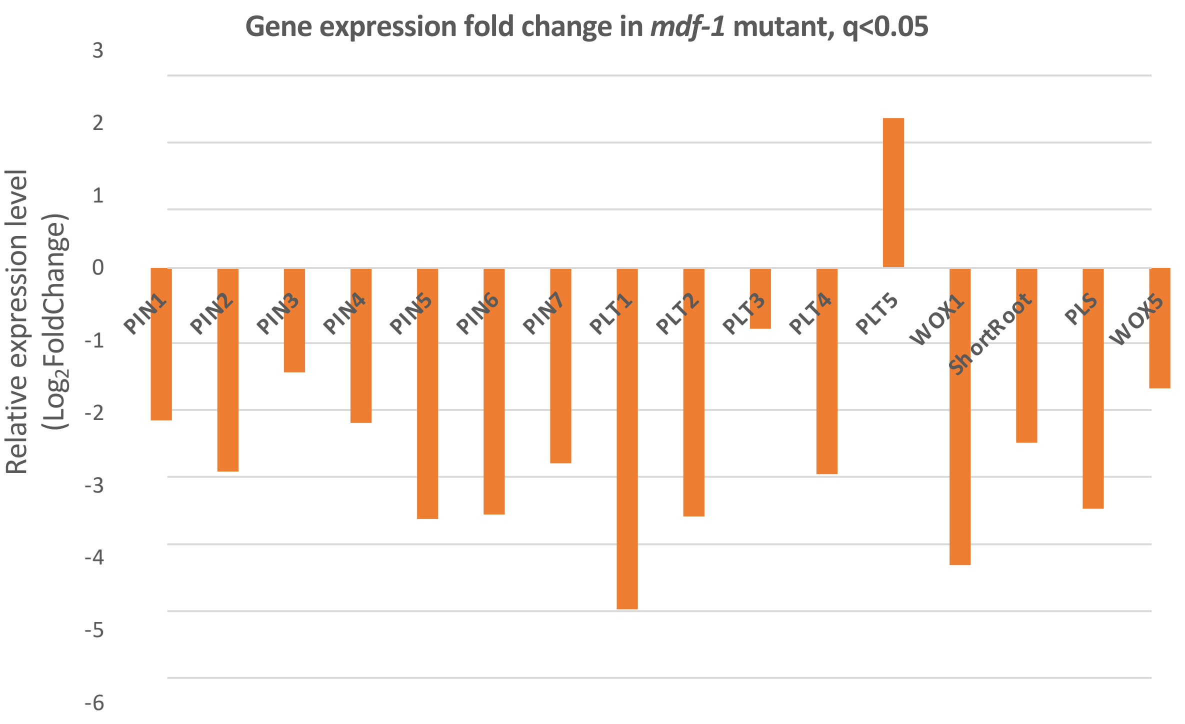

Fig. S3I. Differential expression analysis (DeSeq2) of genes required for root meristem development and auxin transport in *mdf-1* compared to Col-0. Except for *PLETHORA5* (*PLT5*), all are down-regulated in *mdf-1*.

Fig. S4

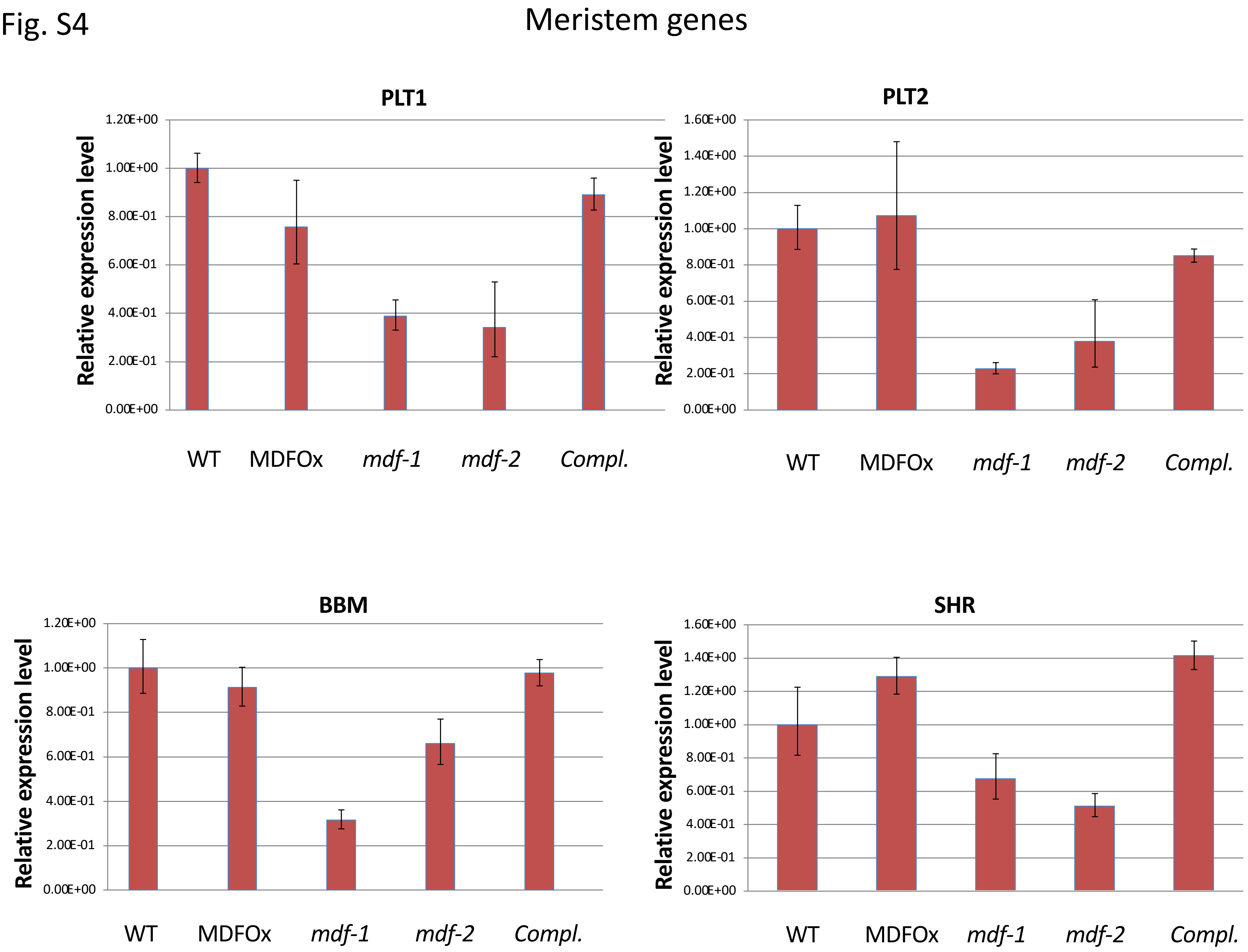

Development • Supplementary information

Fig. S4

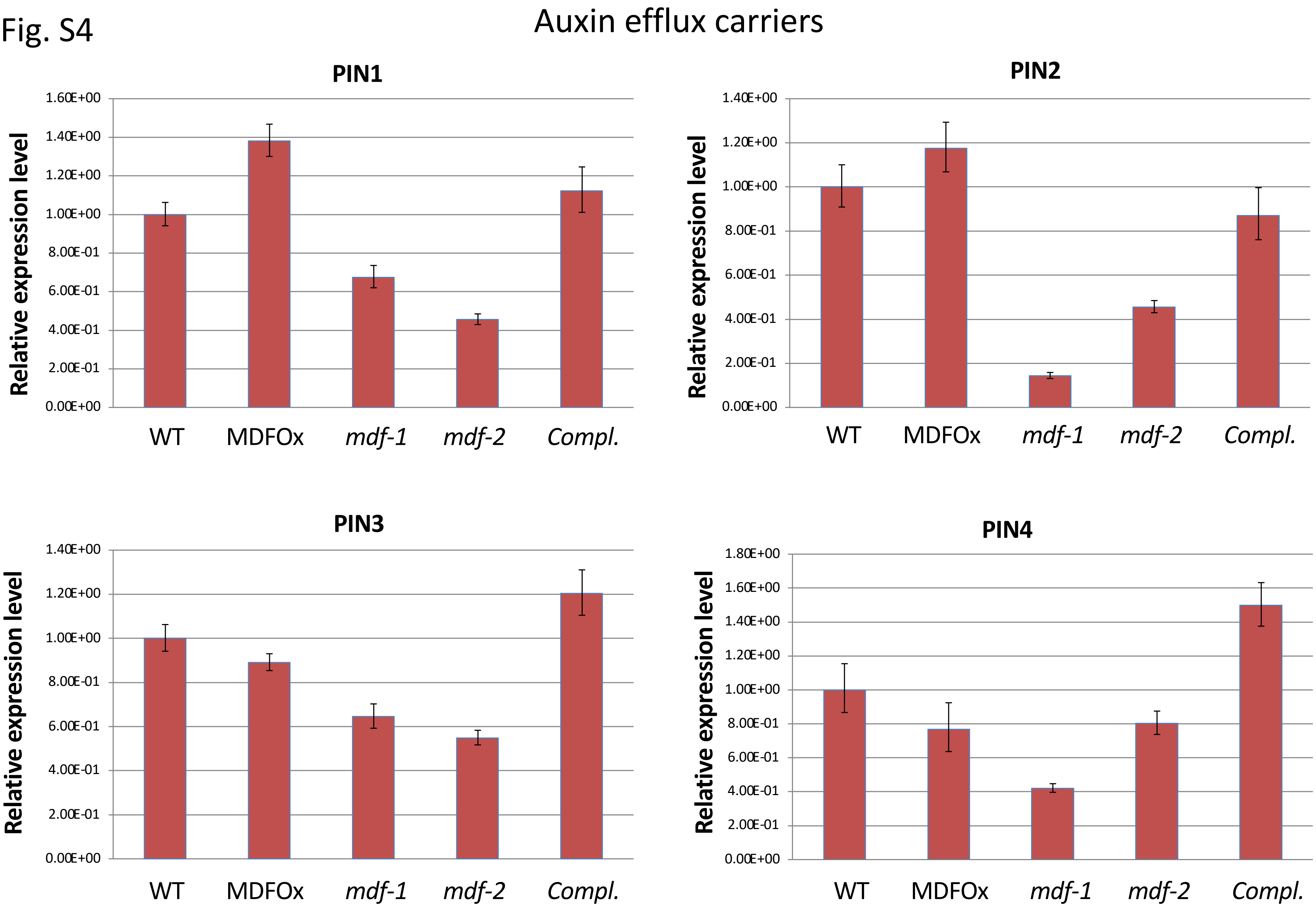

Development • Supplementary information

Fig. S4

Auxin efflux and response genes

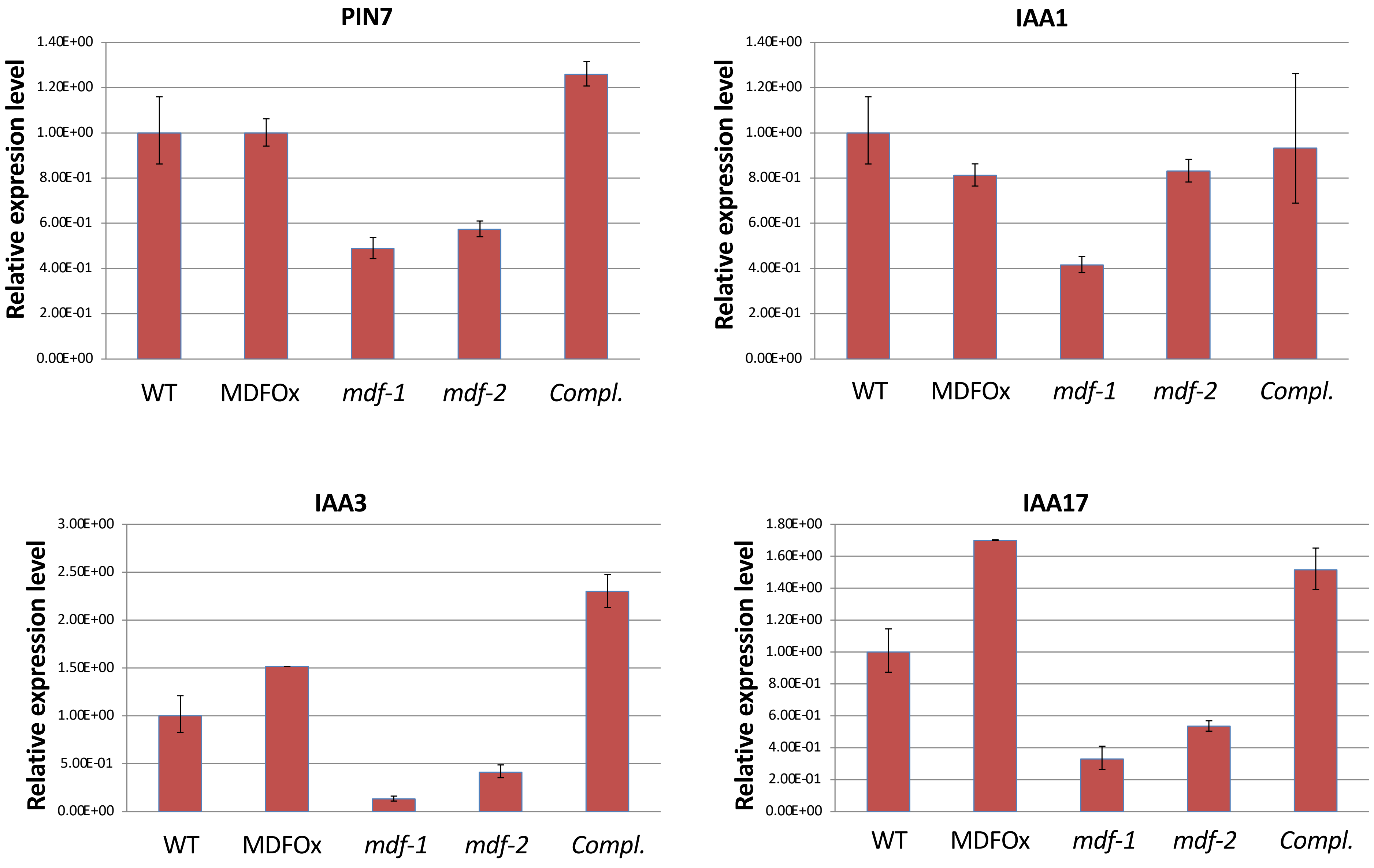

Development • Supplementary information

Fig. S4

U SnRNPRNA processing genes

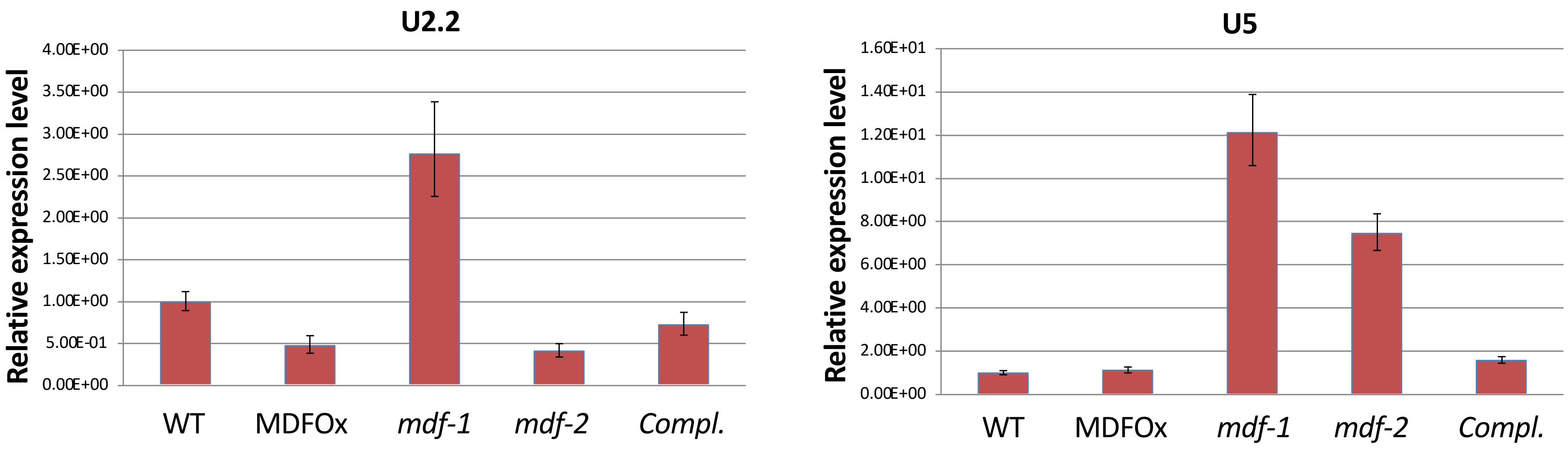

Fig. S4

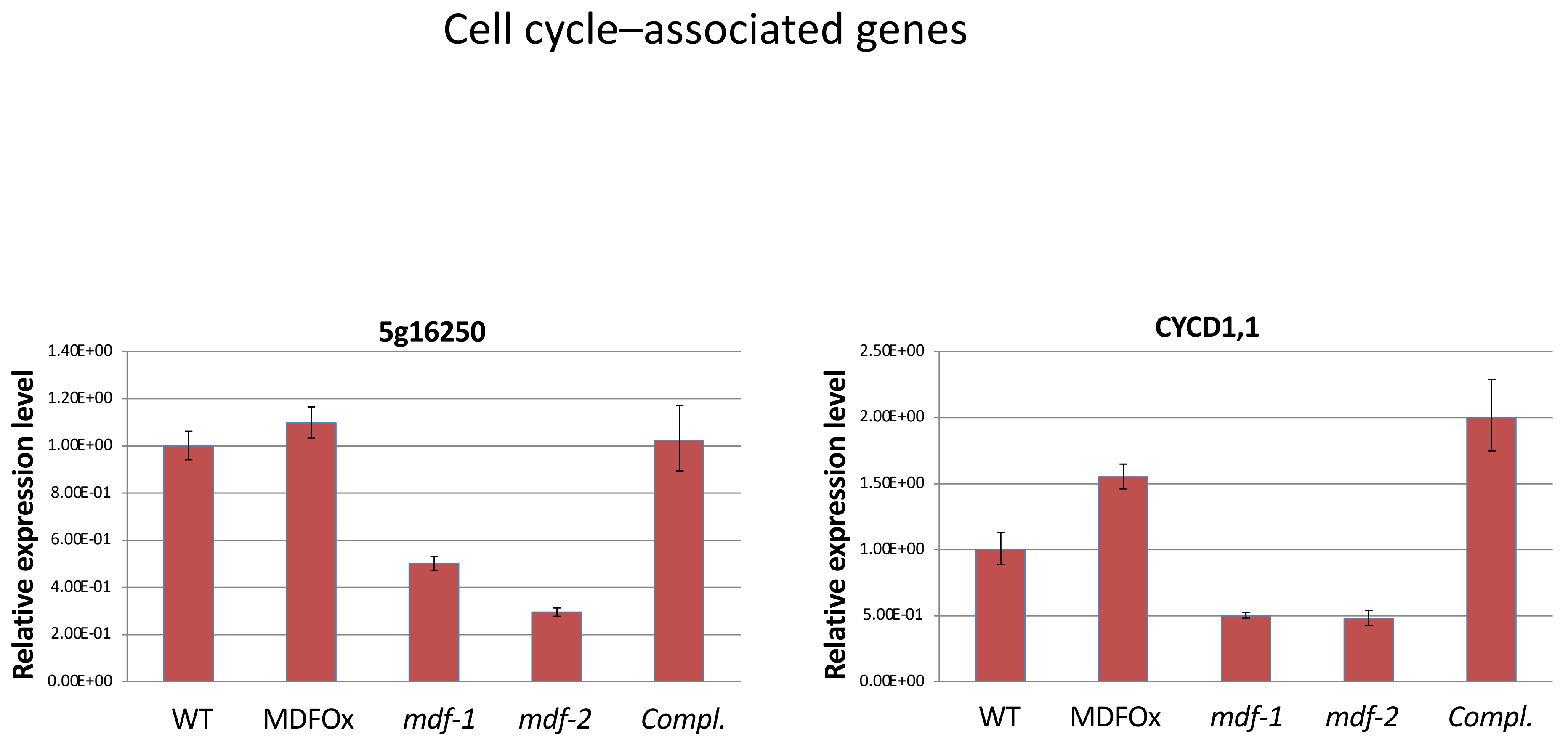
Cell cycle–associated genes
5g16250
Relative expression level
1.40E+00
1.20E+00
1.00E+00
8.00E-01
6.00E-01
4.00E-01
2.00E-01
0.00E+00
WT
MDFOx
mdf-1
mdf-2
Compl.
CYCD1,1
Relative expression level
2.50E+00
2.00E+00
1.50E+00
1.00E+00
5.00E-01
0.00E+00
WT
MDFOx
mdf-1
mdf-2
Compl.

Fig. S4

Stress response genes

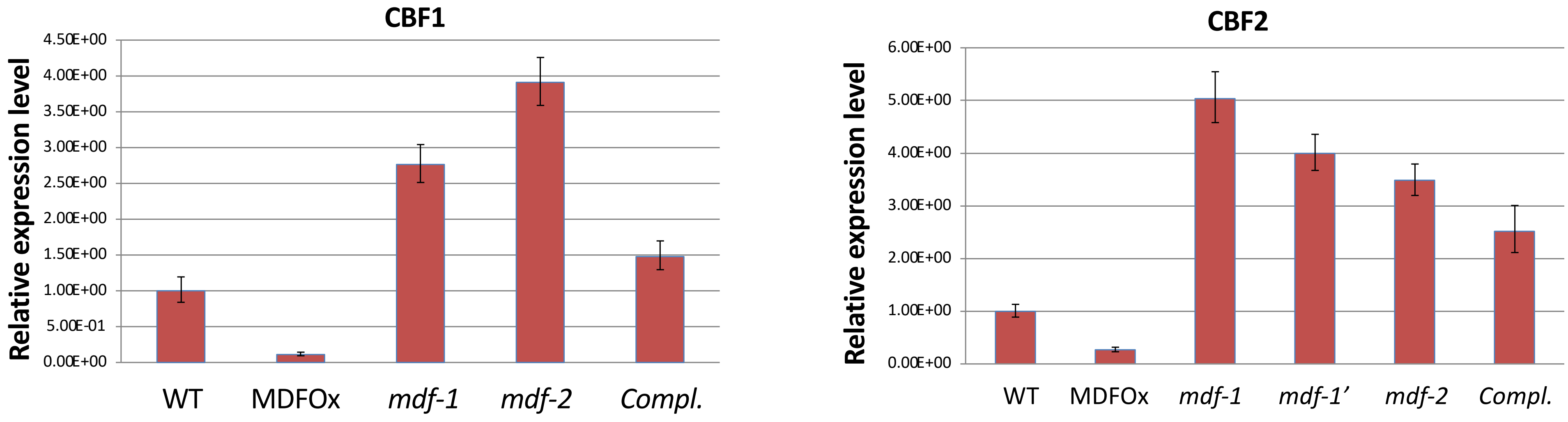

Fig. S4. Expression of selected meristem and stress-related genes in *mdf* mutants and *MDF* overexpressers

qRT-PCR analysis of selected genes involved in meristem function, auxin transport and response, U SnRNPRNAprocessing, cell cycle and stress response in wild-type (Col-0), transgenic MDF over expressing (*pro35S::MDF*), *mdf-1* and *mdf-2* mutant and *mdf-1* mutant complemented with a *proMDF::MDF* gene fusion at 7 d.p.g.

Development • Supplementary information

RSZ33 – splicing factor

Retained intron disrupts reading frame and inserts a PTC eventually. No zinc finger or RS domain are translated correctly

Protein sequence

```
1 MPRYDDRYGN TRLYVGRLSS RTRTRDLERL FSRYGRVRDV DMKRDYAFVE
51 FGDPRDADDA RHYLDGRDFD GSRITVEFSR GAPRGSRDFD SRGPPPGAGR
101 CFNCGVDGHW ARDCTAGDWK NKCYRCGERG HIERNCKNSP KKLRRSGSYS
151 RSPVRSRSPR RRRSPSRSLS RSRSYSRSRS PVRRRERSVE ERSRSPKRMD
201 DSLSPRARDR SPVLDDEGSP KIIDGSPPPS PKLQKEVGSD RDGGSPQDNG
251 RNSVVSPVVG AGGDSSKEDR SPVDDDYEPN RTSPRGSESP
```

C-X2-C-X4-H-X4-C zinc finger domain

RRRSPSRSLS RSRSYSRSRS – RS domain (protein binding)

Fig. S5. A. RSZ33 protein sequence showing zinc finger and RS domains, and site of retained intron that creates a premature termination codon (PTC).

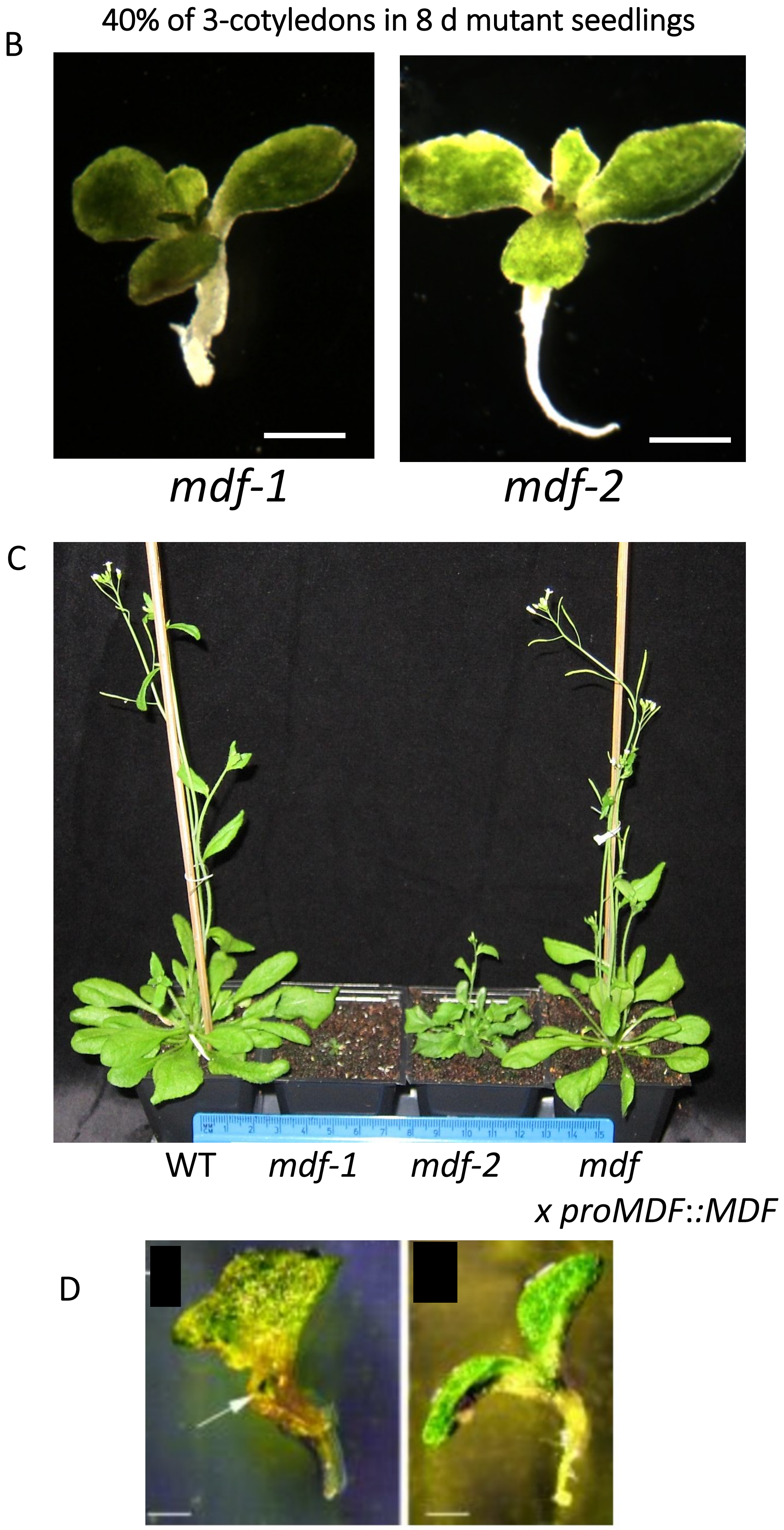

RSZ33 overexpression at 8 d seedling stage
(with permission from Kalyna et al. 2003)

Fig. S5

Fig. S5. *mdf* mutants and *RSZ33* overexpressers have similar phenotypes Phenotypes of (B) *mdf-1* and *mdf-2* seedlings at 8 d.p.g., (C) wild-type (Col-0), *mdf-1* and *mdf-2* mutant and *mdf-1* mutant complemented plants at 4 weeks post-germination; and (D) *RSZ33* transgenic overexpressers at 8 d.p.g. Scale bar = 0.5 cm.

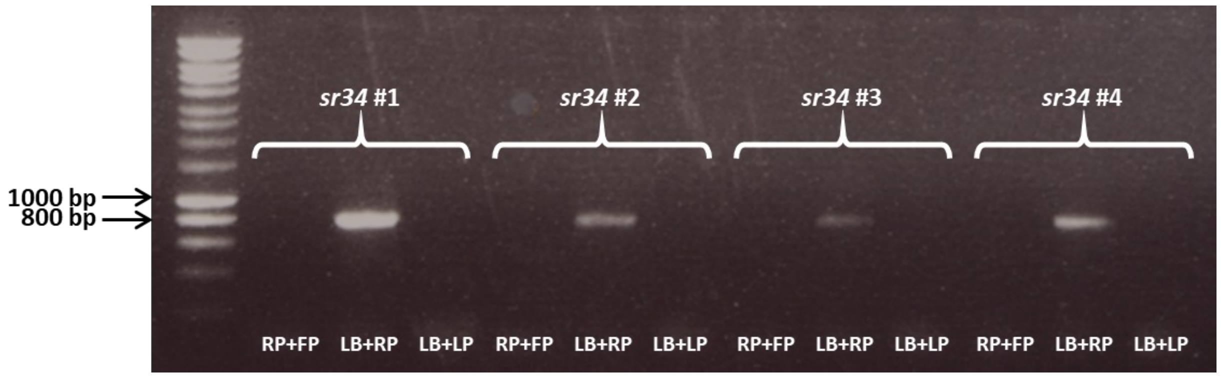

Fig. S6. Verification of *sr34* SALK T-DNA insertion mutants.
Genotyping of 4 individuals (#1-4). All mutants were found to be homozygous for the T-DNA insertion since amplification was only seen with the gene-specific RP and the insertion-specific LB, with a band in the correct size range of 610-910 bp. No band was seen when both gene-specific primers, LP and RP, were used, indicating no amplification of the WT *SR34* gene. The T-DNA insertion can be assumed to be in the orientation predicted by the SIGnAL database, since no bands were present when the LP and the LB primer were used. PCR amplifications with the same 3 combinations of primer pairs were conducted using WT gDNA (not shown) as a control, where amplification was only seen with the gene-specific LP and RP primers. Hyperladder™ 1kb (Bioline) was used as the DNA size marker on the left hand side.

Development • Supplementary information

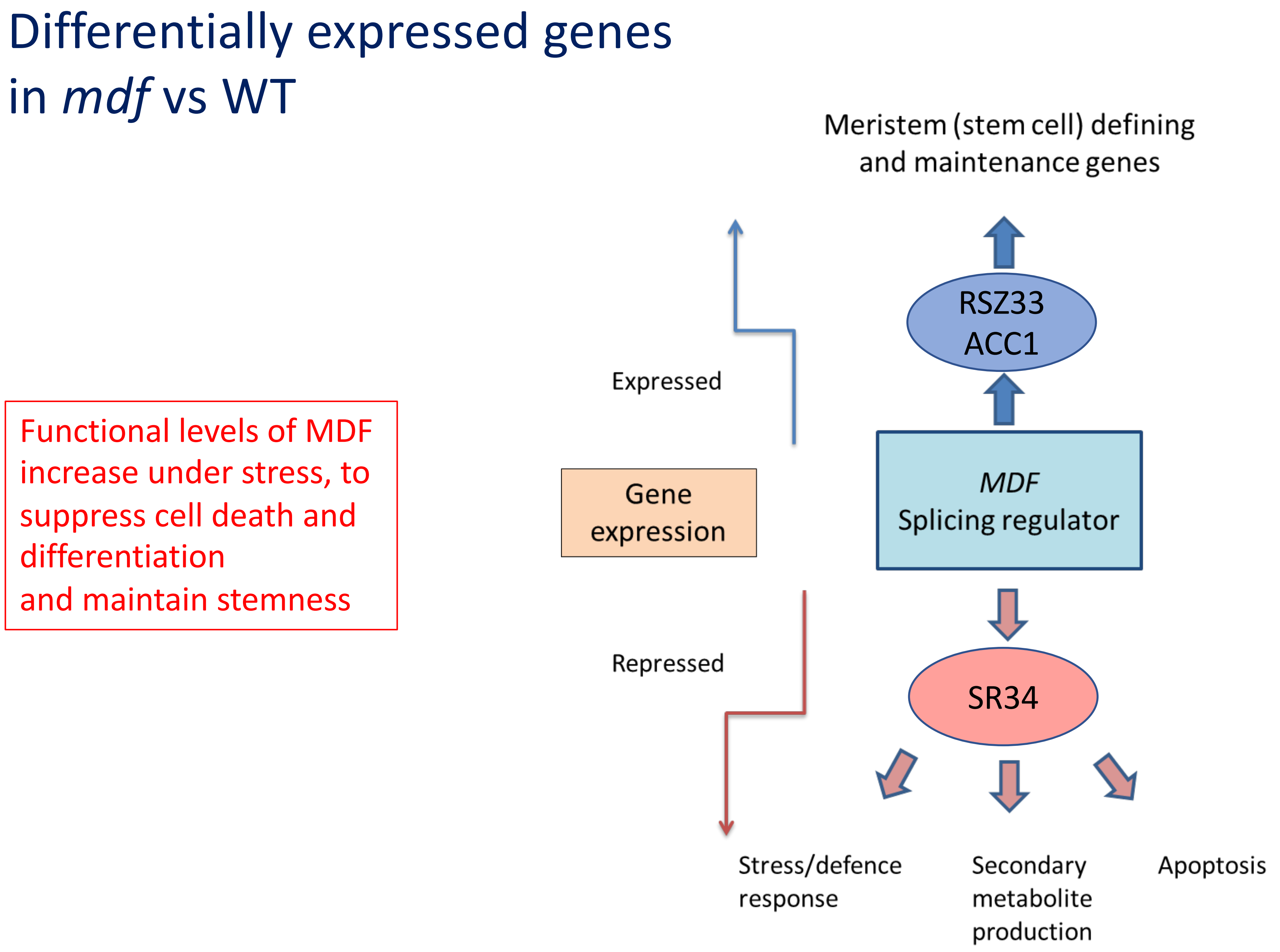

Fig. S7. Role of MDF in control of stemness and cell differentiation Model describing the relationship between MDF and the antagonistic processes of stem cell maintenance and differentiation, stress and cell death. Functional levels of MDF increase under stress to suppress cell death and differentiation, such as through the splicing target *SR34*, and to maintain stem cell identity and cell division activity in the meristem, such as through the splicing targets *RSZ33* and *ACC1*.

Table S1. RNA-seq data showing up- and down-regulated expression levels of selected meristem genes of interest for *mdf-1* and *mdf-2*.

Click here to download Table S1

Table S2. AtRTD2 DeqSeq data showing fold-change transcript levels in *mdf-1* compared to wildtype at fdr of 0.05.

Click here to download Table S2

Table S3. Primers

Click here to download Table S3
